# Integrating single Ni sites into biomimetic networks of covalent organic frameworks for selective photoreduction of CO_2_†
†Electronic supplementary information (ESI) available: Experimental details and characterization. See DOI: 10.1039/d0sc01747g


**DOI:** 10.1039/d0sc01747g

**Published:** 2020-06-09

**Authors:** Xin Chen, Qiang Dang, Rongjian Sa, Liuyi Li, Lingyun Li, Jinhong Bi, Zizhong Zhang, Jinlin Long, Yan Yu, Zhigang Zou

**Affiliations:** a Key Laboratory of Eco-materials Advanced Technology , College of Materials Science and Engineering , Fuzhou University , Fuzhou 350108 , China . Email: lyli@fzu.edu.cn ; Email: yuyan@fzu.edu.cn ; Email: zgzou@nju.edu.cn; b Institute of Oceanography , Ocean College , Minjiang University , Fuzhou , Fujian 350108 , China; c State Key Laboratory of Photocatalysis on Energy and Environment , College of Chemistry , Fuzhou University , Fuzhou 350108 , China; d Eco-materials and Renewable Energy Research Center , College of Engineering and Applied Sciences , Nanjing University , Nanjing 210093 , China

## Abstract

Selective photoreduction of CO_2_ into a given product is a great challenge but desirable. Inspired by natural photosynthesis occurring in hierarchical networks over non-precious molecular metal catalysts, we demonstrate an integration of single Ni sites into the hexagonal pores of polyimide covalent organic frameworks (PI-COFs) for selective photoreduction of CO_2_ to CO. The single Ni sites in the hexagonal pores of the COFs serve as active sites for CO_2_ activation and conversion, while the PI-COFs not only act as a photosensitizer to generate charge carriers but also exert a promoting effect on the selectivity. The optimized PI-COF with a triazine ring exhibits excellent activity and selectivity. A possible intra- and inter-molecular charge-transfer mechanism was proposed, in which the photogenerated electrons in PI-COFs are efficiently separated from the central ring to the diimide linkage, and then transferred to the single Ni active sites, as evidenced by theoretical calculations.

## Abstract 2

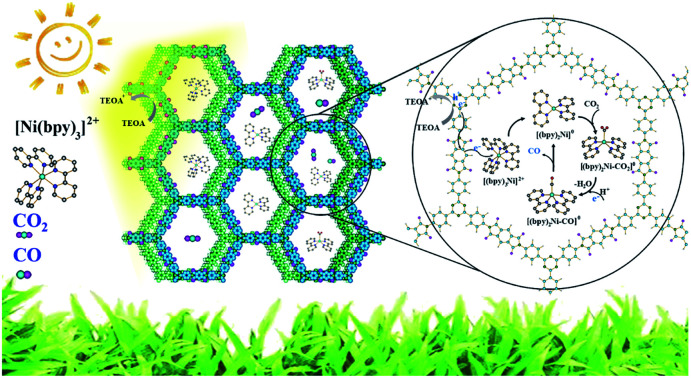
Fabrication of biomimetic photocatalytic systems consisting of PI-COFs and molecular Ni complexes for selective reduction of CO_2_ is demonstrated.

## Introduction

Selective photoconversion of CO_2_ to a target industrial product is an intriguing approach to simultaneously enrich solar energy and utilize CO_2_.^1^,^2^ However, efficient photoreduction of CO_2_ with high selectivity, particularly in aqueous solution, is a considerable challenge because of the multi-electron reaction process and the competing H_2_ evolution in the CO_2_ reduction reaction.^3^,^4^ So far, many molecular metal complex based photocatalytic systems have been developed to selectively reduce CO_2_ into solar fuels with high efficiency. Molecular complexes as precursors of single active sites with tailorable and versatile coordination possess maximum efficiency of catalytic sites in the reaction.^5^–^8^ However, most of the systems suffer from insufficiently stable and expensive photosensitizers to achieve high performance.^9^ The utilization of semiconductors would be a promising alternative approach for photocatalysis,^10^,^11^ as they always possess higher photostability.^12^–^14^ In practical applications, the photocatalytic performance of semiconductor-based catalytic systems is still limited because of their exterior surface catalytic mechanism, always leading to a limited utilization of photogenerated charges. Taking inspiration from nature, where photocatalysis for converting solar energy into chemical energy occurs in the hierarchical networks in plants' leaves with non-precious metal catalysts,^15^,^16^ the combination of single metal sites and a hierarchical porous semiconductor may offer an applicable approach towards the development of photocatalytic systems for selective conversion of CO_2_.^17^ In this context, it is desirable to explore novel semiconductors with intrinsic hierarchical porosity to accommodate single active sites and maximize the transfer of photogenerated charges to the active sites.

The development of covalent organic frameworks (COFs)^18^ provides a promising platform for photocatalysis.^19^–^24^ The periodic and permanent porosity endow COFs with a nature-mimicking architecture, while the diverse compositions and synthetic approaches allow COFs with tunable microstructures and optical and electronic structures.^25^–^29^ The highly conjugated structure in-plane in COFs can ensure the mobility of photoinduced charges.^30^–^33^ Moreover, COFs with tunable porosity can accommodate guest molecules for target applications.^34^–^39^ In terms of catalysis, the microenvironment in the cavity of a heteroatom-rich COF may impose complicated effects on the active sites and its catalytic performance.^40^ Compared to metal–organic frameworks, organic polymers and inorganic networks, COFs possess a metal-free skeleton and periodic porosity to form biomimetic microenvironments as in plants' leaves for the accommodation of metal molecular catalysts. Profiting from the unique characteristics of COFs, it is reasonable to expect the integration of single metal sites in photoactive COFs for photoreduction of CO_2_.

**Fig. 1:**
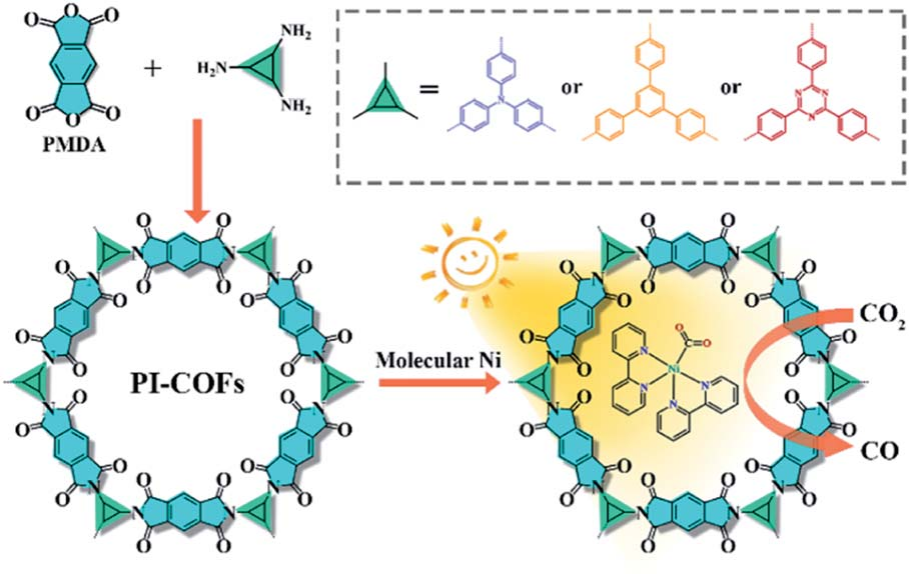
Synthesis of PI-COFs and the schematic illustration of photocatalytic selective reduction of CO_2_ by PI-COFs with a molecular Ni catalyst.

Herein, we report an integration of single Ni sites in the biomimetic channels in polyimide covalent organic frameworks (PI-COFs) for selective photoreduction of CO_2_ to CO (Fig. 1). The excellent catalytic performance mainly arises from the synergistic effects of single Ni sites and the PI-COFs, in which the engineered PI-COFs *via* adjusting the building units not only act as hosts for accommodating single Ni sites but also are responsible for the generation and separation of charge carriers.

## Results and discussion

**Fig. 2:**
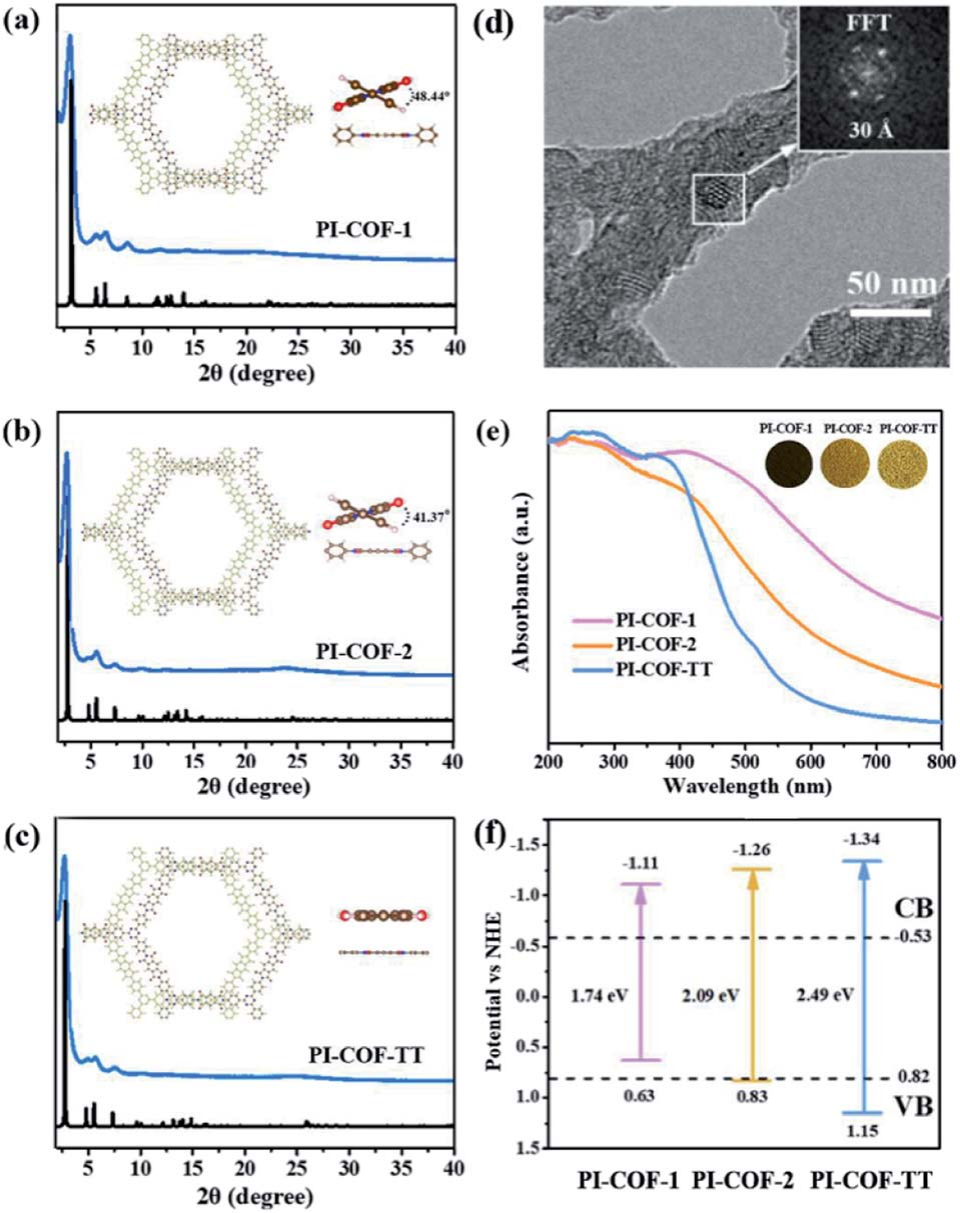
(a–c) PXRD patterns that were experimentally observed (blue) and the simulated pattern (black) of PI-COF-1, PI-COF-2 and PI-COF-TT, respectively. Top inset: the structures of PI-COFs; (d) TEM images of PI-COF-TT; (e) UV-vis diffuse reflectance spectra and (f) band gap structures of PI-COFs.

Three polyimide covalent organic frameworks (PI-COFs) were synthesized by coupling pyromellitic dianhydride (PMDA) with tris(4-aminophenyl)amine (TAPA), 1,3,5-tris(4-aminophenyl)benzene (TAPB) and 1,3,5-tris(4-aminophenyl)triazine (TAPT), respectively, and are denoted as PI-COF-1, PI-COF-2 (ref. 41) and PI-COF-TT,^42^ respectively. The formation of the COFs was assessed by Fourier transform infrared (FT-IR), solid-state ^13^C NMR spectroscopy and powder X-ray diffraction (PXRD). FT-IR spectra of all three COFs showed strong peaks at 1720–1725 cm^–1^ (Fig. S1–S3†), confirming the formation of five-membered imide rings. In the solid-state ^13^C NMR spectroscopy, PI-COFs showed the characteristic signal for the carbonyl carbon of the imide ring at 165 ppm (Fig. S4†).^43^ The overlapping peaks from 116.5 to 144.7 ppm were attributed to phenyl carbons. The PXRD patterns of PI-COFs show prominent peaks, indicating their crystalline nature. The experimental profiles of PI-COFs match well with their simulated PXRD patterns indicating a serrated stacking bnn net with adjacent sheets slipping by 1/4 of the unit cell distances (Fig. 2a–c).^41^ All PI-COFs exhibit similar geometries, whereas the linkages in PI-COF-2 and PI-COF-1 twist rather than remaining planar as in PI-COF-TT, and the phenyl group is tilted by 48.44 and 41.37° with respect to the diimide plane, respectively. The N_2_ adsorption/desorption measurements show that all as-synthesized PI-COFs have a pore size in the range of 1.5 to 3.5 nm calculated using the nonlocal density functional theory (NLDFT), which is in agreement with the pore sizes predicted from the theoretical crystal structures. The Brunauer–Emmett–Teller (BET) surface areas of PI-COF-1, PI-COF-2 and PI-COF-TT are calculated to be 475, 1175 and 825 m^2^ g^–1^, respectively (Fig. S5–S7†). All three PI-COFs exhibit good CO_2_ uptake behavior due to the polarity of the polymer surface^44^ (Fig. S8–S10†), and PI-COF-TT shows the highest isosteric heat (*Q*_st_) (29.76 kJ mol^–1^) for CO_2_ adsorption at low coverage (Fig. S11†). This may be attributed to the dipole–quadrupole interactions between CO_2_ molecules and the imide groups and triazine rings in the framework.^44^,^45^ Scanning electron microscopy (SEM) images show that PI-COF-1 and PI-COF-2 exhibit a “sphere-like” morphology (Fig. S12†), while PI-COF-TT is a cross-linked network (Fig. S13†). Crystalline domains were identified in PI-COF-TT in the transmission electron microscopy (TEM) images (Fig. 2d). The presence of regular lines with a spacing of ∼3.0 nm in TEM images was consistent with the interatomic distances of the [100] plane inferred from PXRD and computational models. TEM images of PI-COF-1 and PI-COF-2 showed that the dense nanospheres were comprised of small nanoparticles (Fig. S14†). PI-COFs are stable in common organic solvents, water and acidic aqueous solutions (Fig. S15 and S16†). Thermogravimetric analysis (TGA) showed that PI-COFs can be stable up to 450 °C in argon (Fig. S17†), and no obvious changes of crystal intensity were observed after heating to 300 °C (Fig. S18†).

UV-vis diffuse reflectance spectra (DRS) show that all three PI-COFs absorb light in the ultraviolet and parts of the visible region (Fig. 2e), suggesting the optical band gaps of 1.74, 2.09 and 2.49 eV for PI-COF-1, PI-COF-2 and PI-COF-TT (Fig. S19†), respectively. The intrinsic absorption band edge varies with the central ring of the COFs. From adjusting triazine, phenyl and tertiary amine units as the central ring, the electron-rich property of the central ring increased, so a remarkable red-shifted absorption of PI-COFs was observed. The band structures of the as-synthesized PI-COFs were obtained from the combination of optical absorption spectra and Mott–Schottky plots (Fig. S20–S22†), and are shown in Fig. 2f. Obviously, the changes of the electronic and steric properties of the central ring result in a progressively enlarged band gap and the corresponding conduction band (CB) and valence band (VB) positions. It is noteworthy that the reduction potentials of the CB electrons in PI-COFs are much more negative than the reduction potentials of CO_2_ to various hydrocarbon fuels, making them suitable for reduction of CO_2_.

**Fig. 3:**
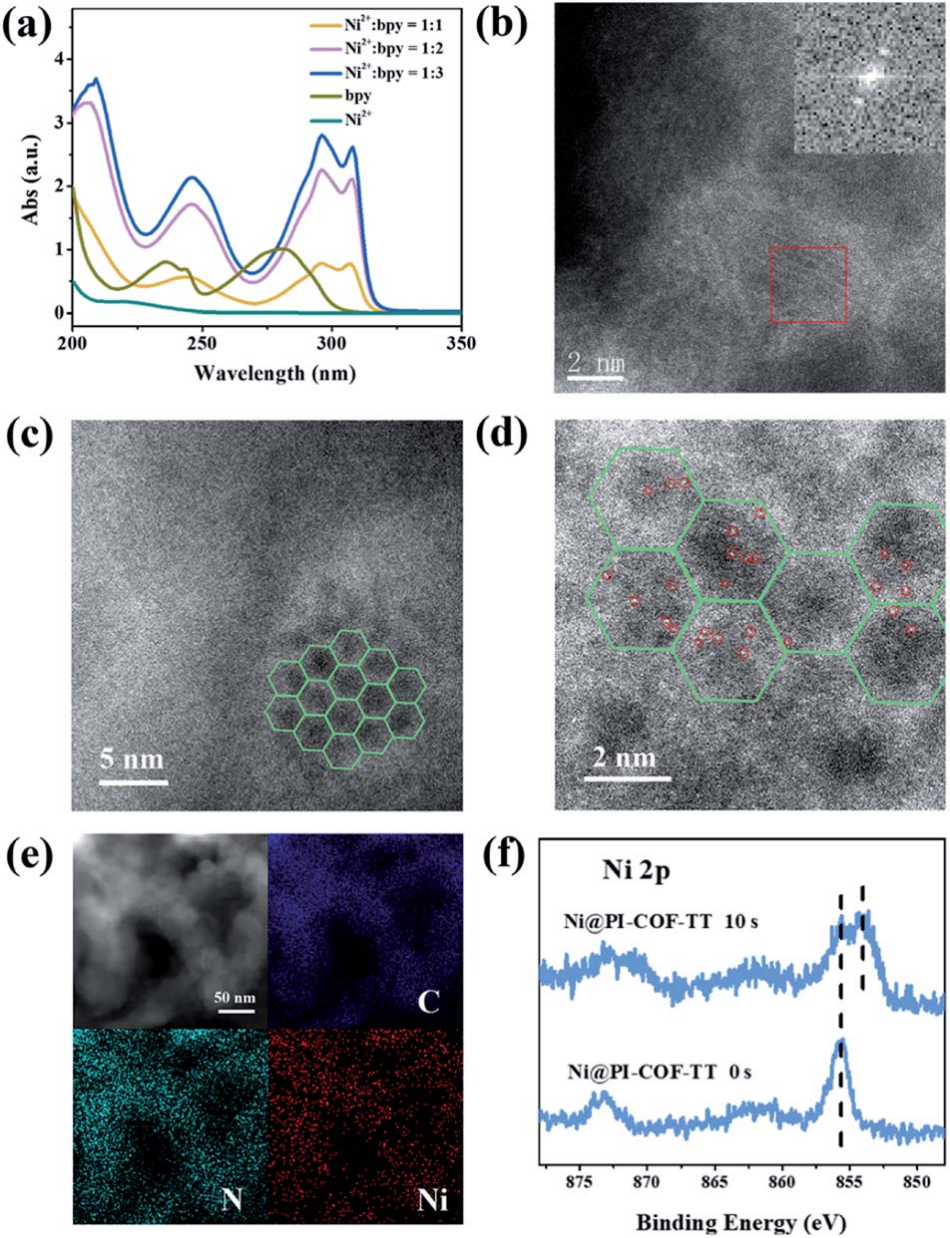
(a) The UV-vis absorption spectra of the *in situ* formed [Ni(bpy)_3_]^2+^ by the consecutive addition of bpy to a solution of Ni[(ClO)_4_]_2_ in acetonitrile. (b–d) Aberration-corrected HAADF-STEM images of Ni@PI-COF-TT, and the FFT image is shown in the inset in (b). (e) EDX elemental mapping image of Ni@PI-COF-TT; (f) Ni 2p XPS upon Ar^+^ sputtering of Ni@PI-COF-TT.

Although Re-modified COFs have been previously employed in photocatalytic reduction of CO_2_,^46^,^47^ it is desirable to develop non-precious metal-based catalytic systems.^48^ The [Ni(bpy)_3_]^2+^ complex has been shown to reduce CO_2_ actively in electrocatalysis^49^ or in homogeneous photocatalysis.^50^ The combination of photoactive COFs and [Ni(bpy)_3_]^2+^ as photocatalytic systems may offer a viable approach for conversion of CO_2_. The photocatalytic activity of PI-COFs toward reduction of CO_2_ in aqueous solution was studied by using self-assembled [Ni(bpy)_3_]^2+^ (bpy is 2,2′-bipyridyl) as a precursor of active sites. The [Ni(bpy)_3_]^2+^ complex can be easily formed upon addition of a bpy ligand and Ni(ii) salt as evidenced by UV-vis absorption spectra. A remarkable red-shift of bpy in UV-vis absorption spectra indicates the coordination of bpy with Ni ions (Fig. 3a). The encapsulation of molecular Ni complexes into the COFs (denoted as Ni@PI-COF-TT) was confirmed by aberration-corrected high-angle annular dark-field scanning transmission electron microscopy (HAADF-STEM), energy dispersive X-ray (EDX) mapping, X-ray photoelectron spectroscopy (XPS), PXRD and N_2_ sorption measurements. HAADF-STEM images clearly show the crystalline domain in Ni@PI-COF-TT (Fig. 3b and c). The fast-Fourier transform (FFT) image confirms the crystalline nature of Ni@PI-COF-TT. Evidently, the bright spots corresponding to the Ni single sites are distributed in the hexagonal pores of Ni@PI-COF-TT (Fig. 3d), and not on the external surface of the stacking layers. EDX elemental mapping and TEM images reveal that Ni ions are uniformly dispersed in the framework (Fig. 3e and S23†). XPS investigations with Ar-ion etching were conducted on Ni@PI-COF-TT. The peak at 856.0 eV was assigned to Ni^2+^ (Fig. 3f). After Ar^+^ etching, the intensity of the characteristic peaks of Ni^2+^ relatively increased, further confirming the encapsulation of the molecular Ni complex in the channels of Ni@PI-COF-TT. A new peak at 854.1 eV is attributed to the reduction of Ni^2+^ to Ni^0^, which was caused by Ar ion bombardment. Compared to PI-COF-TT, the PXRD peak of Ni@PI-COF-TT at 2.7° corresponding to the (110) facet weakened, due to the disorder induced by the Ni complexes inside the channels of Ni@PI-COF-TT (Fig. S24†). The PXRD peak intensity can be recovered after the removal of [Ni(bpy)_3_]^2+^ from the COF by washing with water. The BET surface area of Ni@PI-COF-TT is obviously reduced yet can be recovered after [Ni(bpy)_3_]^2+^ removal (Fig. S25†). The reduction of porosity in Ni@PI-COF-TT is mainly attributed to the occupancy of molecular Ni complexes in the channels.

**Fig. 4:**
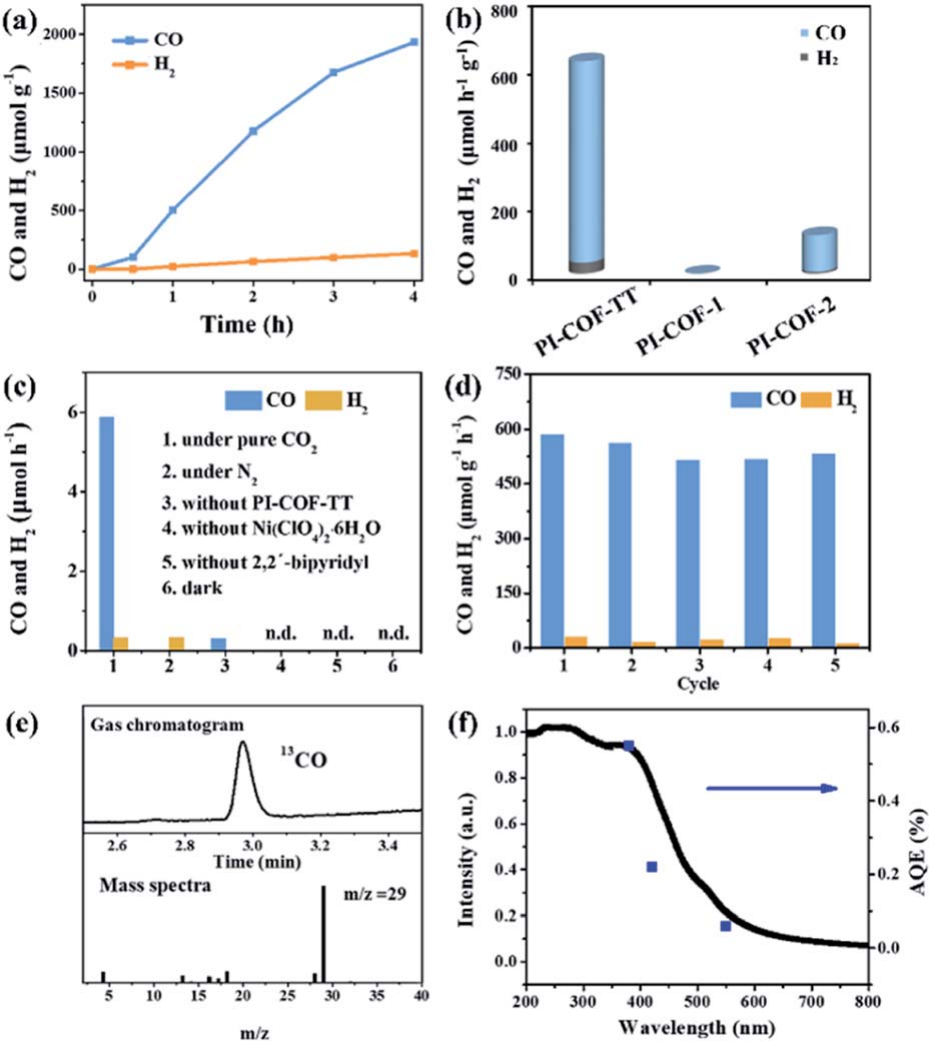
(a) Kinetic profile of CO production by PI-COF-TT; (b) catalytic performance of PI-COFs; (c) control experiments using PI-COF-TT in 2 h CO_2_ photoreduction; (d) recycling test for CO_2_ reduction by PI-COF-TT in 2 h; (e) GCMS measurements of the gas production from ^13^CO_2_ photoreduction; (f) wavelength dependence of CO evolution in a 1 h reaction.

**Fig. 5:**
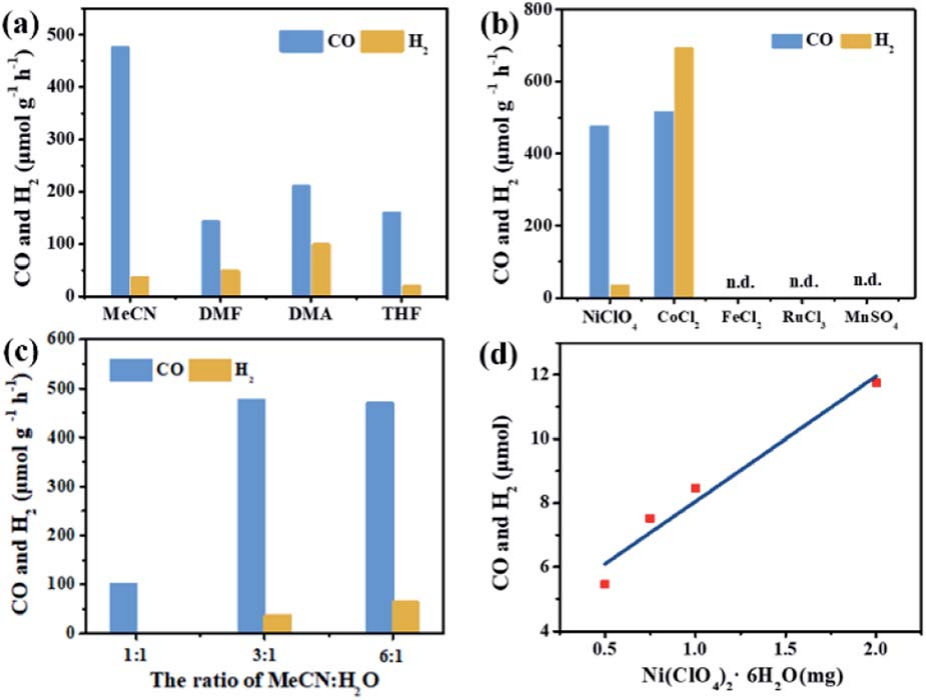
Effect of solvents (a), metal salts (b), H_2_O concentration (c) and Ni concentration (d) on CO_2_ reduction over PI-COF-TT in a 2 h reaction.

In the photocatalytic CO_2_ reduction reaction, under the optimized reaction conditions, PI-COF-TT can generate 1933 μmol g^–1^ CO with a 93% selectivity over H_2_ production in a 4 h reaction (Fig. 4a), while PI-COF-1 and PI-COF-2 showed relatively low catalytic activities (Fig. 4b). It is noteworthy that the activity of PI-COF-TT is lower than that of the previously reported Ni-TpBpy-based catalytic system, mainly as a result of the absence of precious metal sensitizers. Nevertheless, the catalytic performance of PI-COF-TT is among the best compared to other noble-metal-free catalytic systems that have been reported so far (Table S1†). Control experiments show that the photocatalytic reduction of CO_2_ results from the coexistence of PI-COF-TT and [Ni(bpy)_3_]^2+^ (Fig. 4c). Cycling experiments indicate the catalytic and structural stability of PI-COF-TT in the photocatalysis (Fig. 4d and S26†). To confirm the origin of the as-formed CO, ^13^CO_2_ labelling experiments were performed. A major signal at a mass/charge ratio of 29 on the spectrum corresponding to ^13^CO appears, confirming that the generated CO comes from the reduction of CO_2_ (Fig. 4e). There were no detectable hydrocarbon products such as HCOOH and CH_3_OH when analyzing the reaction solvent through ^1^H NMR and high-performance liquid chromatography (HPLC) (Fig. S27†). The trend of CO production matches well with the optical absorption spectrum of PI-COF-TT (Fig. 4f), suggesting that the CO_2_ reduction is indeed induced by PI-COF-TT. The quantum efficiency (AQE) of PI-COF-TT was estimated to be 0.55% at 380 nm. Acetonitrile was found to be a favourable reaction solvent for CO_2_ reduction with a relatively high activity and selectivity (Fig. 5a), mainly because of its appropriate coordination ability to Ni, which can not only contribute to the stabilization of the Ni active sites, but also retain the accessibility to the Ni center. Other metal complexes were tested under similar reaction conditions (Fig. 5b). The results show that the Co complex could also reduce CO_2_ to produce CO efficiently but with a low selectivity for CO compared to H_2_. H_2_O in the reaction could significantly influence the catalytic activity and product selectivity. Upon increasing the amount of H_2_O in the reaction, the production of both CO and H_2_ decreases (Fig. 5c), probably due to the intrinsic hydrophobicity of PI-COFs. With the increment of the amount of Ni^2+^ in the reaction the reduction production gradually increased, further confirming that the Ni single sites act as catalytically active sites (Fig. 5d).

**Fig. 6:**
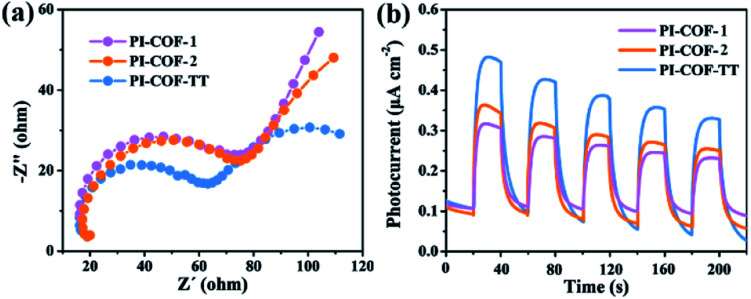
(a) EIS Nyquist plots of bulk PI-COFs; (b) photocurrent responses of PI-COFs.

The photoelectrochemical properties of the PI-COFs were investigated by electrochemical impedance spectroscopy (EIS) and transient photocurrent measurements. Nafion solution was used as an additive to PI-COF powder to form the active layer,^51^ which can form a homogeneous ink with the COF and further help to attach onto the surface of electrodes. Control experiments showed negligible effects of Nafion on the current density of electrodes (Fig. S28†). As shown in Fig. 6a, Nyquist curves show that all three PI-COFs exhibit two semicircles. The small semicircles in the high-frequency region correspond to the charge transfer resistance, while the large ones in the low-frequency region are related to diffusion resistance. The radius of the semicircles in the high frequency region of PI-COF-TT is smaller than that of other two PI-COFs, suggesting that the triazine ring in the PI-COFs obviously improves the rate of charge transfer.^52^ Linear potential sweep measurements of PI-COF-TT showed a higher current density of 10.35 μA cm^–2^ at 0.1 V *vs.* RHE compared to PI-COF-1 and PI-COF-2 (Fig. S29†). The current density is directly correlated with the increase in the catalytic performance. In addition, PI-COF-TT exhibits higher photocurrent intensity than PI-COF-1 and PI-COF-2 (Fig. 6b), which probably contributes to the improved photocatalytic activity.^53^ To show the morphology of the active photosensitizer layer, SEM of the PI-COF electrodes was performed. Top view SEM images revealed that the FTO glass electrodes can be well covered by PI-COFs (Fig. S30†). All PI-COF deposits show interstitial voids and textural porosity which may also contribute to their catalytic performance.^54^,^55^

**Fig. 7:**
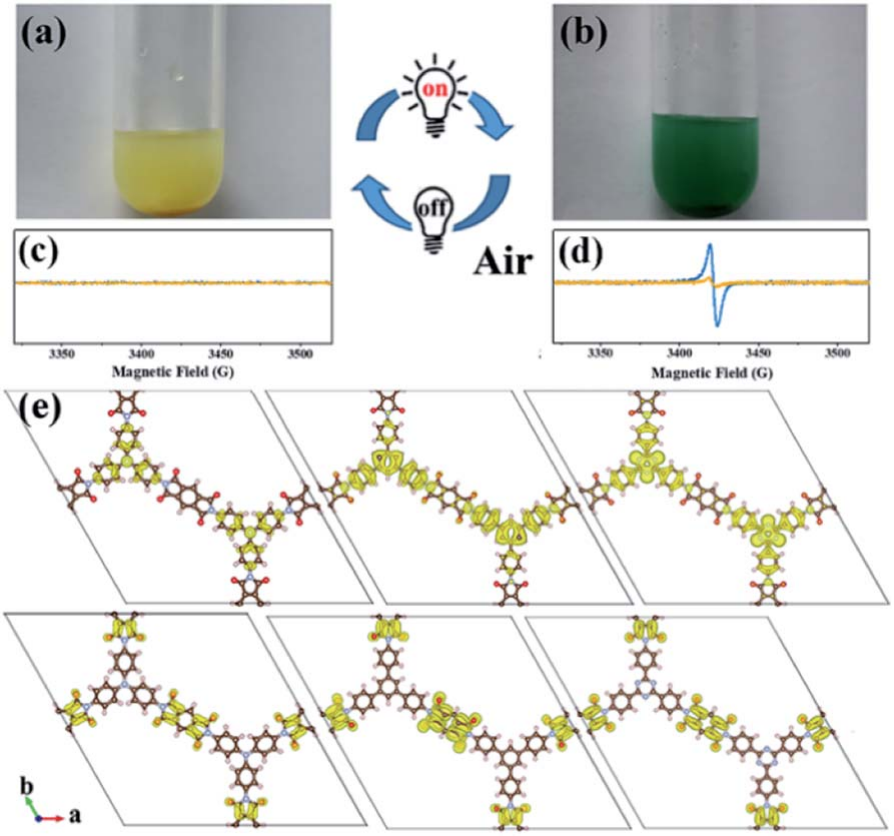
Photos and corresponding EPR spectra of PI-COF-TT (blue) and Ni@PI-COF-TT (yellow) with TEOA in acetonitrile solution before (a and c), and after (b and d) 10 min light illumination; charge density distribution for PI-COF-1, PI-COF-2 and PI-COF-TT at the CB minimum and the VB maximum (e) (drawn at the isosurface level of 0.0001 e Å^–3^).

An interesting photochromic phenomenon was observed in the photoreduction of CO_2_ over PI-COFs (Fig. S31†). When the catalytic system was irradiated under a CO_2_ atmosphere, as shown in Fig. 7a and b, the color of PI-COF-TT changed from the original yellow to green to yellow to orange. After the introduction of air into the reaction, the color of PI-COF-TT changed from orange back to the original yellow. The color change of PI-COF-TT takes place in the presence of triethanolamine (TEOA) under light irradiation (Table S2†). The electron paramagnetic resonance (EPR) spectrum of the suspension of PI-COF-TT and TEOA in acetonitrile shows a prominent increment of EPR signals after light illumination (Fig. 7c and d), indicating charge generation upon photoexcitation,^56^ and leading to the photochromic phenomenon. In comparison with PI-COF-TT, the EPR signal intensity of Ni@PI-COF-TT decreased predominantly under identical conditions, revealing the electron transfer from PI-COF-TT to [Ni(bpy)_3_]^2+^. The solid-state photoluminescence (PL) emission spectra of PI-COFs show negligible peaks around 400–700 nm compared to the strong peak of PMDA at about 430 nm (Fig. S32–S34†). The weak emission of PI-COFs mainly results from the intramolecular charge transfer from the central rings acting as the electron donor to pyromellitic diimide units acting as the electron acceptor in PI-COFs.^31^,^57^ Cyclic voltammetry (CV) and optical tests of pyromellitic diimide, *N*,*N*′-bis(phenyl)pyromellitimide, triphenylamine, triphenylbenzene and triphenyltriazine as model compounds of building blocks were performed to reveal their relative energy levels (Fig. S35†). The LUMO levels of *N*,*N*′-bis(phenyl)pyromellitimide are very close to those of triphenylamine, triphenylbenzene and triphenyltriazine, which means that the connection of pyromellitic diimide and triphenylamine, triphenylbenzene or triphenyltriazine affording an extended conjugation framework could presumably contribute to the electron transfer. When a physical mixture of pyromellitic diimide and triphenyltriazine was employed in the catalytic reaction, no reduction products such as CO, CH_4_ and H_2_ were observed (Fig. S36†), which further reveals the vital role of the formation of the conjugated structure in PI-COFs for charge transfer. Density functional theory (DFT) calculations show that the CB and VB wave functions of PI-COFs are separately localized on the diimide units and the central rings, respectively (Fig. 7e). Due to their narrow band gaps, the PI-COFs can be excited to form electron–hole pairs by light irradiation. The photogenerated electrons transfer from the central rings to the diimide units. The planar π-conjugated structure in PI-COF-TT brings a superior separation of photogenerated electrons and holes compared to PI-COF-1 and PI-COF-2,^58^ thus contributing to the enhanced catalytic efficiency.

**Fig. 8:**
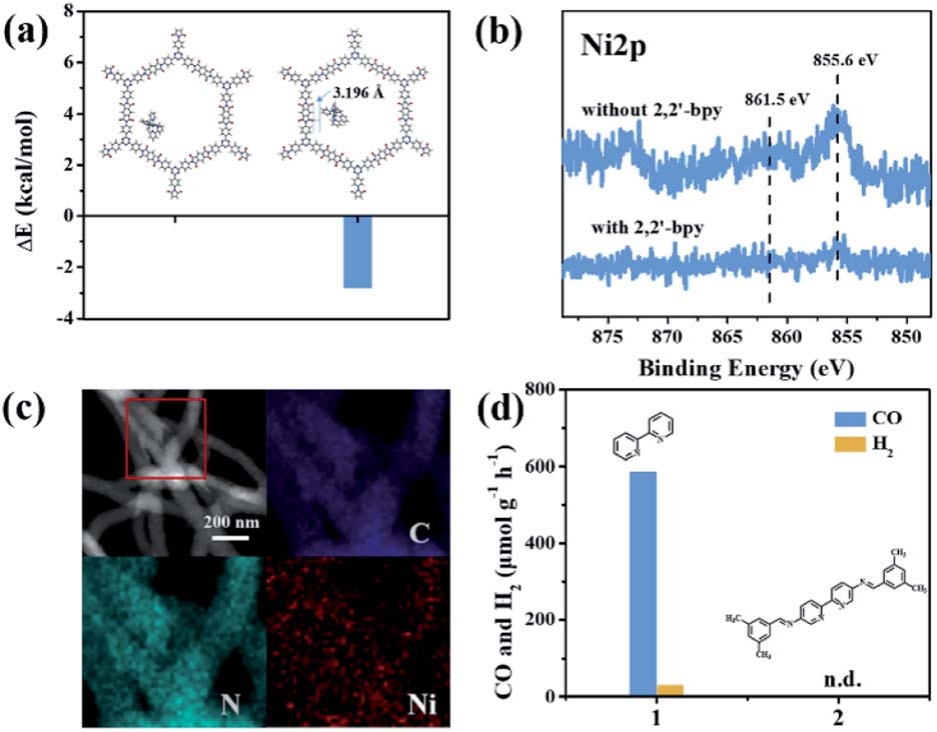
(a) The adsorption energy (Δ*E*) of Ni(bpy)_3_^2+^ in PI-COF-TT. The distance between the Ni complex and the framework (inset); (b) Ni 2p XPS of the recovered PI-COF-TT with and without 2,2′-bipyridyl from the catalytic reaction; (c) EDX elemental mapping of the recovered PI-COF-TT from the photoreduction of CO_2_ with 2,2′-bipyridyl; (d) 2,2′-bipyridyl (1) and modified 2,2′-bipyridyl (2) performance in the photoreduction of CO_2_ over PI-COF-TT in 2 h.

It is noteworthy that an obvious induction period accompanies the photochromic phenomenon. Thus, DFT calculations and control experiments were carried out to further explore the reaction mechanism. The *in situ* formed molecular Ni complexes preferred to be adsorbed to the diimide unit and not to the triazine ring, as evidenced by DFT calculations (Fig. 8a). However, the distance between the Ni complexes and framework (3.196 Å) indicates only weak interactions between PI-COF-TT and Ni complexes. The relatively weak signals of Ni 2p XPS (Fig. 8b) and EDX elemental mapping (Fig. 8c and S37†) of the recovered PI-COF-TT with bpy from the catalytic reaction than those of the recovered PI-COF-TT from the reaction without bpy confirmed the weak interactions between the molecular Ni active sites and the framework. PI-COFs with direct impregnation of [Ni(bpy)_3_]^2+^ showed significantly lower activity than PI-COFs with *in situ* formed [Ni(bpy)_3_]^2+^ (Fig. S38†). In the *in situ* formed [Ni(bpy)_3_]^2+^ case, the assembly of Ni ions and the bpy ligand occurred in the pores of PI-COFs, resulting in the uniform distribution of [Ni(bpy)_3_]^2+^ in PI-COFs. However, in the direct impregnation of [Ni(bpy)_3_]^2+^ case, the [Ni(bpy)_3_]^2+^ complexes with a relatively large molecular volume cannot diffuse effectively into the pores of PI-COFs, leading to the inefficient catalytic activity. The recovered PI-COF-TT does not generate the CO product in the additional reaction run in the absence of Ni complexes, further confirming that Ni active sites are free in the channel and only physically adsorbed on the pore surfaces.^59^ Additionally, when a bpy ligand with large substituents was used, the production of CO and H_2_ was not detected under similar reaction conditions (Fig. 8d). This could be due to the steric hindrance of the substituent on the bpy ligand, leading to inefficient contact between the Ni center and COF wall. These noncovalent interactions are always considered to be conducive for retaining their intrinsic properties as well as the sufficient interfacial mass transport.^48^,^60^ Thus, the induction period could be mainly ascribed to the collisional electron transfer from the COF to molecular Ni complexes to form molecular Ni active sites,^48^ which is verified by our previous report showing that [Ni(bpy)_2_]^0^ formed from the reduction of [Ni(bpy)_3_]^2+^ in the photocatalytic reaction serves as the active site for the activation and conversion of CO_2_.^40^ In this case, the molecular [Ni(bpy)_2_]^0^ active sites are produced from the reduction of [Ni(bpy)_3_]^2+^ by photogenerated electrons of PI-COF-TT under UV-vis light irradiation (Fig. S39 and S40†). Since single Ni^0^ atoms are active, excess bpy is necessary to stabilize single Ni sites through the formation of [Ni(bpy)_2_]^0^*via* coordination of bpy to Ni.

**Fig. 9:**
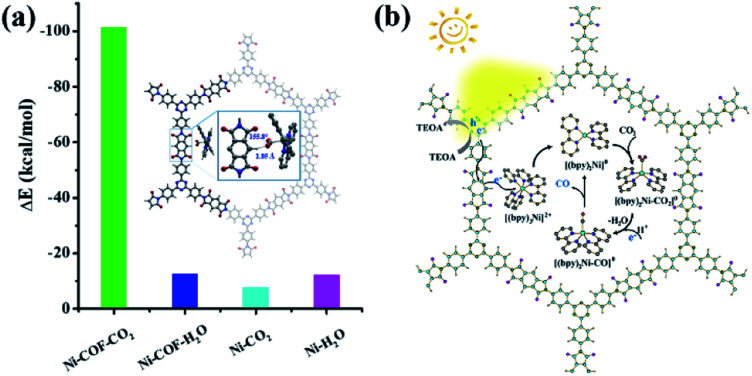
(a) DFT-calculated adsorption energy (Δ*E*, kcal mol^–1^) of CO_2_ and H_2_O on [Ni(bpy)_2_]^0^ with and without PI-COF-TT (inset: the activation of CO_2_ by [Ni(bpy)_2_]^0^ in PI-COF-TT). Ni–COF–CO_2_, Ni–COF–H_2_O, Ni–CO_2_ and Ni–H_2_O are the complexes of [Ni(bpy)_2_]^0^ with CO_2_ and/or H_2_O without and/or with PI-COF-TT, respectively. (b) Proposed reaction mechanism for photoreduction of CO_2_ over PI-COF-TT.

In order to understand the selectivity trends between the CO and H_2_ products, the adsorption energies of CO_2_ and H_2_O onto [Ni(bpy)_2_]^0^ with and without PI-COF-TT were calculated (Fig. S41 and Table S3†). As shown in Fig. 9a, the adsorption energy of CO_2_ on [Ni(bpy)_2_]^0^ in the presence of PI-COF-TT was significantly lower than that of H_2_O, implying the stronger affinity of molecular [Ni(bpy)_2_]^0^ toward CO_2_, which would facilitate the formation of the key intermediate Ni–CO_2_ adducts and afford selectivity for the CO_2_ reduction product rather than the H_2_ product. It is noteworthy that the adsorption energy of CO_2_ on Ni active sites was obviously reduced by PI-COF-TT through the hydrogen bonding interactions between the hydrogen atom in the PI unit and the activated CO_2_ molecule (Fig. 9a, inset). Accordingly, the selective photoreduction of CO_2_ was greatly dependent on the selective adsorption and activation of CO_2_ on the metal active sites and the special reaction microenvironment. The synergistic photocatalytic system containing PI-COFs and single Ni sites facilitates the selective activation of CO_2_ and inhibits the competitive H_2_ evolution, leading to the enhanced catalytic activity and selectivity.

Based on the above results, a possible mechanism for the selective photoreduction of CO_2_ over PI-COF-TT with molecular Ni complexes was proposed (Fig. 9b). Light illumination on PI-COF-TT generates electron–hole pairs. The holes are reductively quenched by TEOA. The electrons move from the triazine ring to the PI unit and subsequently transfer to the accommodated molecular [Ni(bpy)_3_]^2+^ complexes to form [Ni(bpy)_2_]^0^ active sites, which can couple with CO_2_ to produce CO. The open channel of PI-COF-TT facilitates the processes of photogeneration of molecular Ni active sites and the subsequent activation and conversion of CO_2_. Besides, the strong adsorption affinity of PI-COF-TT for CO_2_ may increase the local concentration of CO_2_ in the channels, thereby promoting the formation of Ni–CO_2_ adducts and facilitating the selective reduction of CO_2_.

## Conclusions

In summary, we demonstrated a design of an integration of PI-COFs with Ni single sites for selective photoreduction of CO_2_. The electronic properties of PI-COFs can be facilely tuned for photocatalysis. The photogenerated electron–hole pairs in PI-COFs under irradiation can be efficiently separated through an intra- and inter-molecular charge-transfer mechanism to drive the reduction of CO_2_. The excellent catalytic performance of the COF-based catalytic system mainly arises from the synergistic effects of the photoactive PI-COF and single Ni sites, in which the PI-COF with a nature-mimicking architecture serves as the functionalized host for single Ni sites to promote the reaction. This work presents an integration of Ni single sites in PI-COFs for CO_2_ reduction and a deep understanding of the electron-transfer mechanism in COFs. Based on the diversity of COFs and molecular metal complexes, we believe, inspired by our work, there would be more functionalized COFs developed for robust and efficient catalytic systems for sustainable energy conversion.

## Experimental

### Synthesis of PI-COF-TT

TAPT (35.1 mg, 0.10 mmol) and PMDA (32.7 mg, 0.15 mmol) were placed in a mixed solution of mesitylene/NMP/isoquinoline (0.5 mL/0.5 mL/0.05 mL). The tube was flash frozen at 77 K (liquid N_2_ bath) and degassed by pump–thaw three times. The tube was sealed and heated at 200 °C for 5 days, giving a yellow precipitate. The precipitate was purified by Soxhlet extraction using tetrahydrofuran overnight, and finally dried under vacuum at 80 °C to give PI-COF-TT (yield 66%).

### Photocatalytic reduction of CO_2_

A mixed solution of acetonitrile, H_2_O and triethanolamine (TEOA) (3 : 1 : 1, 5 mL) containing PI-COFs (10 mg), Ni(ClO_4_)_2_·6H_2_O (2 mg, 5.5 μmol) and 2,2′-bipyridyl (15 mg, 0.1 mmol) was purged with CO_2_ for 15 min. The solution was then irradiated under UV-Vis light (300 W Xe lamp, PLS-SEX 300/300UV, 780 mW cm^–2^) at 313 K. After each reaction time, the generated gas in the headspace of the reaction vessel was sampled with a gas-tight syringe and determined by gas chromatography (Agilent 7890B) with both TCD and FID detectors. H_2_ was detected using a TCD detector. CO was converted to CH_4_ in a methanation reactor and then analyzed using an FID detector. The apparent quantum efficiency (AQE) at 380 nm was calculated by the following equation: AQE = (2 × amount of CO molecules evolved in 1 h/number of incident photons in 1 h) × 100%.

## Conflicts of interest

There are no conflicts to declare.

## Supplementary Material

Supplementary information
