# Supplementary material for: Integrating single Ni sites into biomimetic networks of covalent organic frameworks for selective photoreduction of CO2
Source: Chem Sci. 2020 Jun 9;11(26):6915–22. doi: 10.1039/d0sc01747g (PMC7499818; doi:10.1039/d0sc01747g)
Supplement: Supplementary file 1 [file SC-011-D0SC01747G-s001.pdf]

Electronic Supplementary Material (ESI) for Chemical Science.
This journal is © The Royal Society of Chemistry 2020

Supporting Information for

Integrating Single Ni Sites into Biomimetic Networks of Covalent Organic Frameworks for Selective Photoreduction of CO_2

Xin Chen, [‡a] Qiang Dang, [‡a] Rongjian Sa,[b] Liuyi Li, [*a] Lingyun Li, [a] Jinhong Bi,[a, c] Zizhong Zhang,[c] Jinlin Long,[c] Yan Yu, [*a] Zhigang Zou[*ad]

[a]Key Laboratory of Eco-materials Advanced Technology, College of Materials Science and Engineering, Fuzhou University, Fuzhou 350108 (China)

[b]Institute of Oceanography, Ocean College, Minjiang University, Fuzhou, Fujian 350 108 (China)

[c]State Key Laboratory of Photocatalysis on Energy and Environment, College of Chemistry, Fuzhou University, Fuzhou 350108, China.

[d]Eco-materials and Renewable Energy Research Center, College of Engineering and Applied Sciences, Nanjing University, Nanjing 210093 (China)

Table of Contents

Experimental Procedures.........2

Computational Details.........3

Characterization.........5

Supplementary Figures.........7

Calculated Geometries Parameters.........30

References.........67

Experimental Procedures

1. Material Synthesis

Synthesis of functional bpy: 5,5'-diamino-2,2'-bipyridine (46.5 mg, 0.25 mmol) and 3,5-dimethylbenzaldehyde (67 mg, 0.50 mmol) were added to 5 mL of THF and 0.5 mL of 6 M CH_3COOH, and then stirred for 12h in room temperature. Finally, the resultant yellow product was concentrated under reduce vacuum from the reaction solution.

Synthesis of PI-COF-1: TAPA (29.0 mg, 0.10 mmol) and PMDA (32.7 mg, 0.15 mmol) were placed in the mixed solution of mesitylene/NMP/isoquinoline (0.5 mL/0.5 mL/0.05 mL) in a tube. The tube was flash frozen at 77 K (liquid N_2 bath) and degassed by pump-thaw three times. The tube was sealed and heated at 200 °C for 5 days, giving a brown precipitate. The precipitate was purified by Soxhlet extraction using tetrahydrofuran overnight, and finally dried under vacuum at 80 °C to give PI-COF-1 (yield 76 %).

Synthesis of PI-COF-2: TAPB (35.1 mg, 0.10 mmol) and PMDA (32.7 mg, 0.15 mmol) were placed in the mixed solution of mesitylene/NMP/isoquinoline (0.5 mL/0.5 mL/0.05 mL) in a tube. The tube was flash frozen at 77 K (liquid N_2 bath) and degassed by pump-thaw three times. The tube was sealed and heated at 200 °C for 5 days, giving a yellow precipitate. Purification of PI-COF-2 taken by the above method (yield 74 %).

Synthesis of Ni@PI-COF-TT: To a solution of $Ni(ClO_4)_2$•$6H_2O$ (2 mg, 5.5 μmol) and bpy (2.57 mg, 16.5 μmol) in 5 mL of MeCN was added PI-COF-TT (10 mg). The mixture was stirred for 12 hours at room temperature. After the stirring is completed,

the suspension containing PI-COF-TT was evaporated at 60 °C, and dried overnight in vacuo at 60 °C to afford Ni@PI-COF-TT. The samples for TEM and HADDF-STEM analyses were prepared by ultrasonication of the Ni@PI-COF-TT powder in ethanol and directly dropping the dispersion onto a carbon-coated copper grid.

Synthesis of N, N'-bis(phenyl)pyromellitimide: PMDA (30 mg, 0.15 mmol) and aniline (35.1 mg, 0.10 mmol) and were placed in the mixed solution of mesitylene/NMP/isoquinoline (0.5 mL/0.5 mL/0.05 mL) in a tube. The tube was flash frozen at 77 K (liquid N_2 bath) and degassed by pump-thaw three times. The tube was sealed and heated at 200 °C for 10 h, giving a slight yellow precipitate. The precipitate was filtered, washed with water and methanol, and finally dried at 70 °C under vacuum to give slight yellow crystalline solid (yield 78 %). FTIR (KBr, cm^{-1}): 1730 (C=O stretch of imide linkage), 1512 (C=C stretch). It should be noted that the absence of a broad absorption band around 3500-3300 cm^{-1} shows that the monomer is completely imidized. ^{1}H NMR or ^{13}C-NMR spectra could not be recorded due to its low solubility in commen solvents tested.

2. Recyclability test.

The photocatalytic reaction was carried out as the above method for 2 h under UV-Vis light (300 W Xe lamp) at 313 K. After the reaction was completed, the recovered COF-TT was separated by centrifugation, washed with MeCN and ethanol three times and dried under vacuum at 353 K. The recovered COF-TT was used in the next catalytic cycle with the addition of fresh 2,2'-bipyridyl (15 mg, 0.1 mmol) and $Ni[(ClO)_4]_2 \cdot 6 H_2O$ (2.0 mg, 5.5 μmol).

3. Photoelectrochemical measurement.

The photocurrent was recorded with a CHI650E electrochemical workstation (Chen Hua Instruments, Shanghai, China) equipped with a conventional three-electrode cell. The platinum plate electrode and the Ag/AgCl electrode was used as counter electrode and reference electrode, respectively. The working electrodes were prepared as follow: 5 mg sample mixed with 0.5 mL solution (Nafion: DMF=1:2, 1% Nafion in ethanol) were sonicated for 1h to make it dispersible, and then dropping 10 μL of the suspension onto the FTO glass to cover the area of 0.25 cm^2. The electrodes were immersed in 0.2 M Na_2SO_4 aqueous solution which severed as electrolyte solution. Visible-light irradiation was provided by a xenon lamp (300 W) with a 420 nm cut-off filter to illuminate the working electrode. The samples for SEM analyses were prepared by ultrasonication of 5 mg sample in a 0.5 mL solution (Nafion: DMF=1:2) for 1h to make it dispersible, and then dropping the suspension onto FTO glassy electrode.

The Mott– Schottky plots and electrochemical impedance spectroscopy (EIS) plots were measured by a ZAHNER IM6 electrochemical workstation in the similar process to the photocurrent measurement. The Mott–Schottky analysis was carried out in a 0.2 M Na_2SO_4 aqueous solution and the EIS analysis was carried out in a 5 mM $K_3[Fe(CN)_6]$/5 mM $K_4[Fe(CN)_6]$/0.1 M KCl mixed aqueous solution, respectively.

Cyclic voltammetry (CV). CV experiments were performed with a CHI650E electrochemical analyzer in a three-electrode electrochemical cell with a scan rate of 0.1 V s^{-1}. The experiments were conducted in anhydrous dichloromethane with

tetrabutylammonium hexafluorophosphate (0.1 M) as supporting electrolyte and sample (0.1 mM). The auxiliary electrode was a platinum wire. The reference electrode was based on the Ag/AgCl electrode. The working electrode was carbon glass electrode.

Cyclic voltammograms of ferrocene (internal standard) in tetrabutylammonium hexafluorophosphate (0.1 M) in dichloromethane at room temperature showed that $E(Fc/Fc^{+})$ onset was 0.49 V vs Ag/AgCl.

Linear sweep voltammograms (LSV). LSV experiments were performed with a CHI650E electrochemical analyzer in a three-electrode electrochemical cell with a scan rate of 0.01 V s^{-1}. The experiments were conducted in 0.1 M Na_2SO_4 aqueous solution. The auxiliary electrode was a platinum wire. The reference electrode was based on the Ag/AgCl electrode. The working electrodes were prepared as follow: A suspension of 2 mg sample in 0.5 mL Nafion solution (1% in ethanol) were sonicated for 30 min to make it dispersible, and then dropping 20 μL of the suspension onto the carbon glass electrode. The potential vs RHE was calibrated as $E_{RHE} = E_{Ag/AgCl} + 0.61$.

Computational Details.

The optimization of three PI-COFs was performed on the projected augmented wave[1] formalism of DFT via the VASP package[2]. We used generalized gradient approximation with a Perdew-Burke-Ernzerhof (PBE)[3] form for the exchange-correlation functional. A cutoff energy of 400.0 eV was used, and the Brillouin-zone integration was sampled using a (4 × 4 × 2) Monkhorst–Pack mesh. The total energy was converged to 10^{-5} eV. All the atomic positions were optimized until the force tolerance

on each atom was less than 0.01 eV/Å. The supercell of 2 × 2 × 1 was adopted for further properties calculation.

We further used Dmol3 package[4,5] to evaluate the absorption properties of CO_2 and H_2O. The exchange–correlation term was considered using the generalized gradient approximation (GGA) proposed by the Perdew, Burke, and Ernzerhof (PBE)[3], in which the Grimme's DFT-D corrections were adopted. The double numeric quality basis set with polarization functions (DNP)[4,6] was adopted, which was comparable to 6-31G**.[7,8] The numerical basis sets can minimize the basis-set superposition error. A Fermi smearing of 0.005 hartree was utilized. The tolerances of the energy, gradient and displacement convergence were 2×10^{-5} hartree, 4×10^{-3} hartree per Å, and 5×10^{-3} Å, respectively. Herein, the initial structure data of COF was obtained from the former VASP calculations. The isolated system models were taken from a 2 × 2 × 1 supercell with terminal groups replaced as hydrogen atoms. Then only the nickel complex optimized when the PI-COF-TT added and constrained. Subsequently, the constrained calculations were applied during CO_2 and H_2O absorption calculation, only CO_2 and H_2O were relaxed while other species were kept fixed.

The absorption of CO_2 to Ni intensified by the addition of PI-COF-TT, in which one hydrogen bond formed between COF and CO_2 with 1.85 Å length. The angel of O=C=O of CO_2 change from 175.3 to 155.8 when COF added. Additionally, the Ni-C bond length of Ni complex and CO_2 shorten from 3.09 Å to 2.24 Å. The absorption energy of CO_2 to Ni complex in PI-COF-TT was -101.5 kcal/mol, which was more favorable than the H_2O absorbed onto Ni complex in PI-COF-TT process (-12.6 kcal/mol).

Characterization

The starting materials were commercially available and used without further purification. ^1H NMR spectra was recorded on a Bruker AVANCE III NMR spectrometer at 400 MHz, respectively, using tetramethylsilane (TMS) as an internal standard. Solid-state ^{13}C CP/MAS NMR was performed on a Bruker SB Avance III 500 MHz spectrometer with a 4-mm double-resonance MAS probe. FTIR spectra were recorded with KBr pellets using Perkin-Elmer Instrument. Powder X-ray diffraction (XRD) patterns were recorded in the range of $2\theta = 3\text{-}40^o$ on X'pert3 X-ray diffractometer with Cu Kα radiation ($\lambda = 1.5406$ Å). N_2 or CO_2 adsorption and desorption isotherms were measured at 77 K using a Micromeritics ASAP 2020 system. The samples were degassed at 120 °C for 10 h before the measurements. Surface areas were calculated from the adsorption data using Brunauer-Emmett-Teller (BET) equation. The BET surface area was calculated from the range of $0.05<P/P_0<0.25$ in the isotherm. The calculation of the pore size distribution was done using the nonlocal density functional theory (NLDFT) equilibrium model. Field-emission scanning electron microscopy (SEM) was performed on a JEOL JSM-7500F operated at an accelerating voltage of 3.0 kV. The CO gas produced from $^{13}CO_2$ isotope experiments was examined by a gas chromatograph−mass spectrometer (GC-MS, Agilent 7890B-5977B). The equipped column in GC-MS analysis was CP-Molsieve 5A (Agilent Technologies, 25.0 m x 0.32 mm x 30 μm). X-ray photoelectron spectroscopy (XPS) measurements were performed on a Thermo ESCALAB 250 spectrometer, using non-monochromatic Al Kα x-rays as the excitation source and choosing C 1s (284.6 eV) as

the reference line. UV-Vis spectra were recorded using a Agilent Cary 5000 spectrometer. Fluorescence spectra were recorded at room temperature using a FM-4 spectrophotometer, and the slit width for emission was 2 nm. Electron paramagnetic resonance (EPR) measurements were carried out on a Bruker model A300 spectrometer. The liquid phase product was analyzed by a HPLC (Waters e2695). Transmission electron microscope (TEM) was obtained with TECNAI G^2 F20. Aberration-corrected high-angle annular dark-field scanning transmission electron microscopy (HAADF-STEM) was performed on FEI Themis Z.

Supplementary Figure

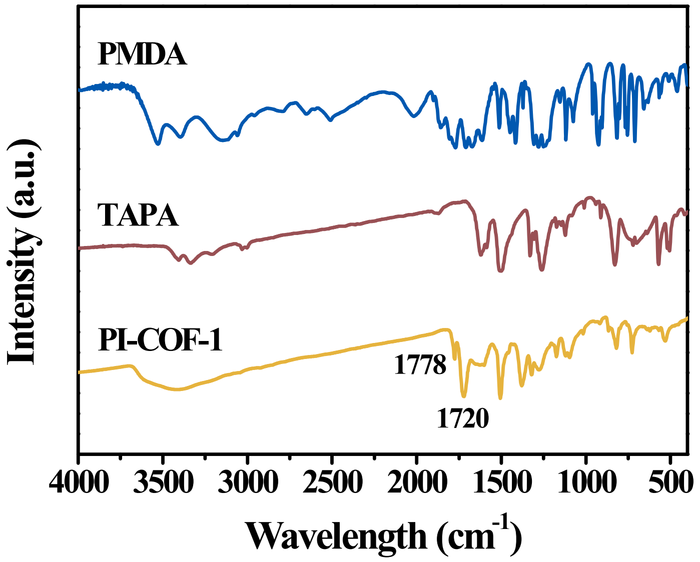

Fig. S1 FT-IR spectra of PI-COF-1.

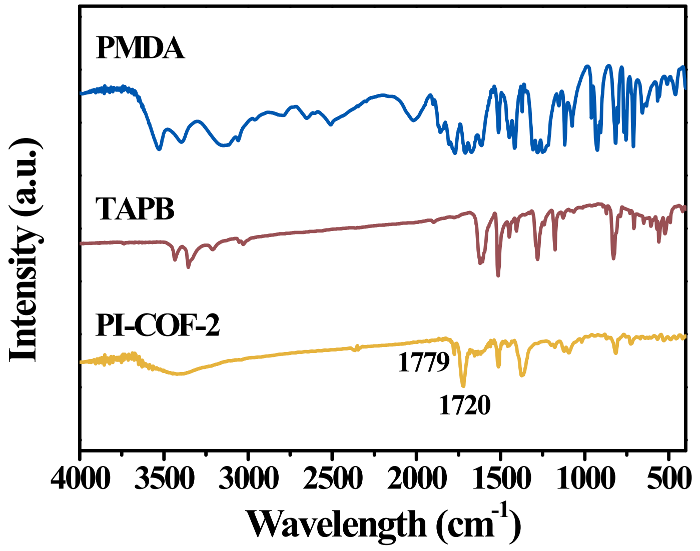

Fig. S2 FT-IR spectra of PI-COF-2.

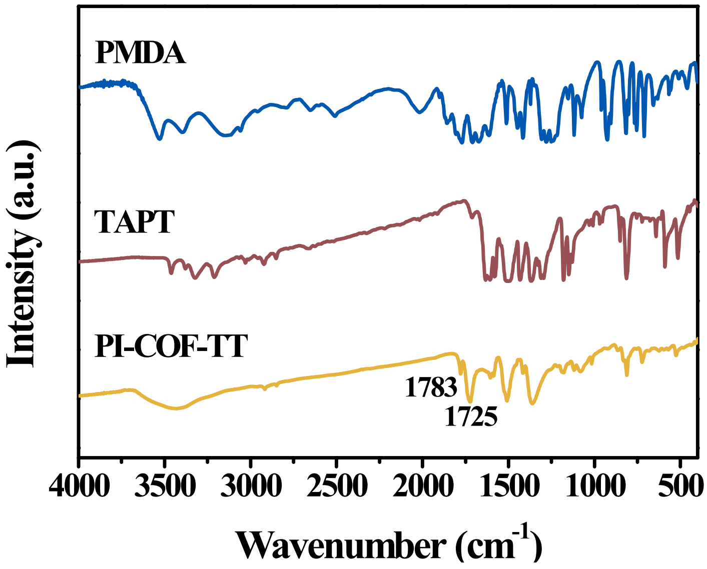

Fig. S3 FT-IR spectra of PI-COF-TT.

FT-IR spectra of PI-COFs showed absorption bands around 1783 and 1720 cm^{-1} for C=O group of the five-membered imide rings. The peaks at 1375-1371 cm^{-1} are assigned to the C-N-C moiety stretching vibration.

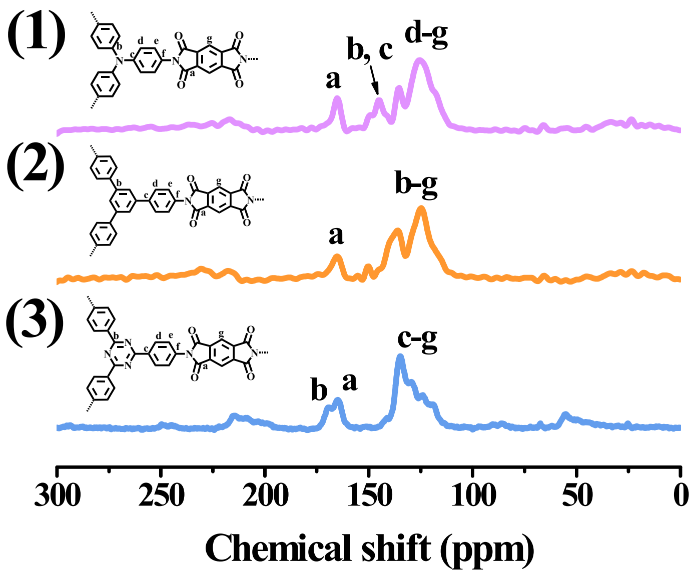

Fig. S4 Solid state ^{13}C NMR spectrum for PI-COF-1 (1), PI-COF-2 (2) and PI-COF-TT (3).

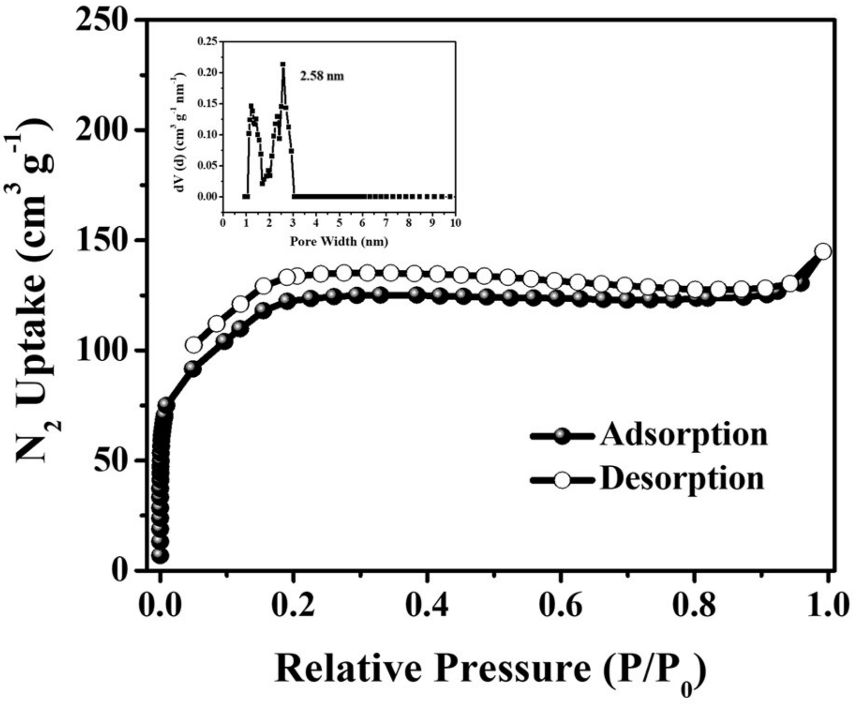

Fig. S5 N_2 sorption isotherms for PI-COF-1 (inset shows the corresponding pore size distribution).

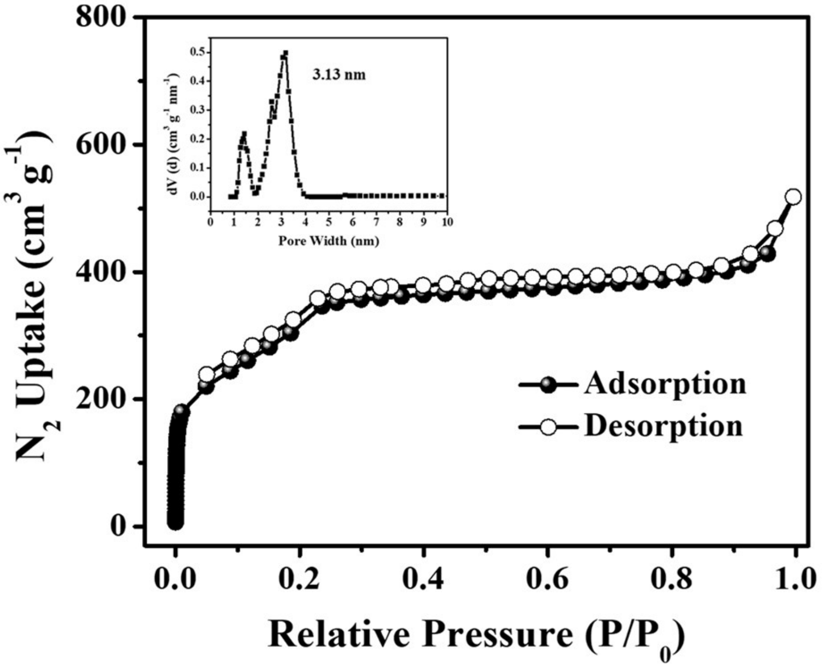

Fig. S6 N_2 sorption isotherms for PI-COF-2 (inset shows the corresponding pore size distribution).

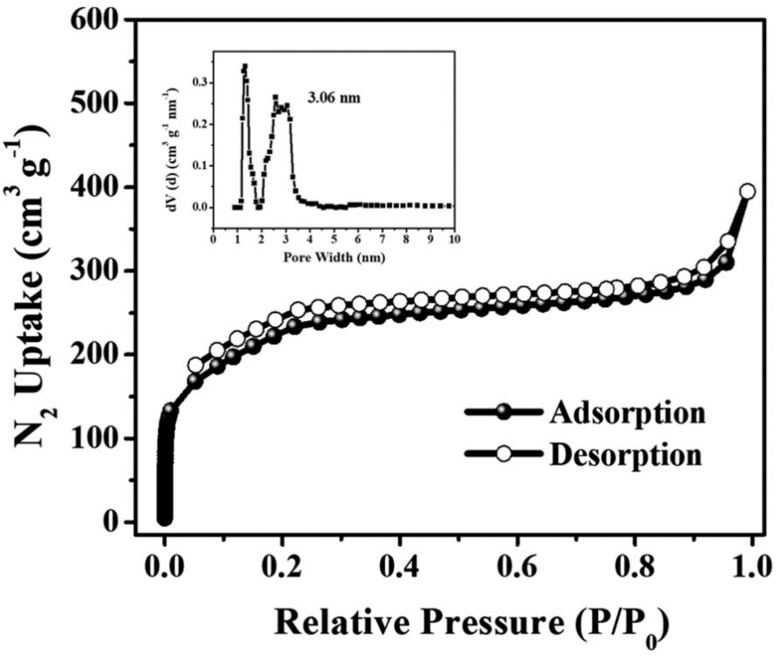

Fig. S7 N_2 sorption isotherms for PI-COF-TT (inset shows the corresponding pore size distribution).

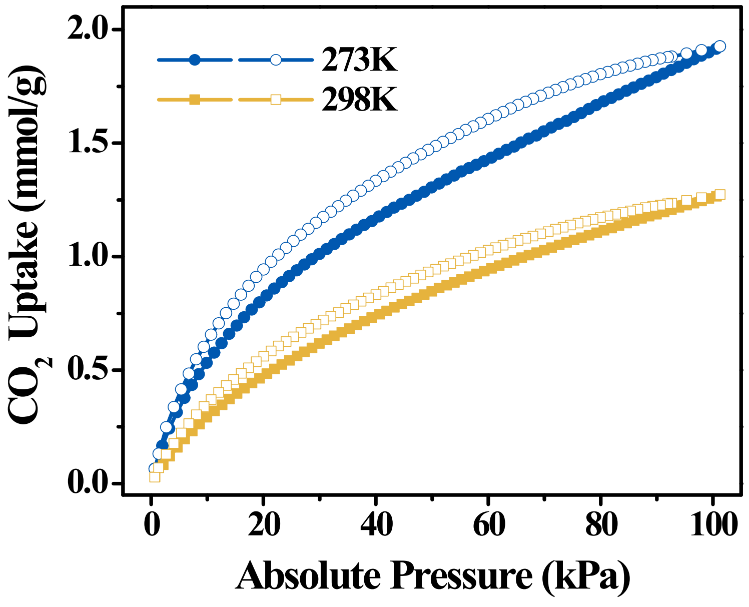

Fig. S8 CO_2 adsorption isotherms for PI-COF-1 at 273K and 298K, respectively.

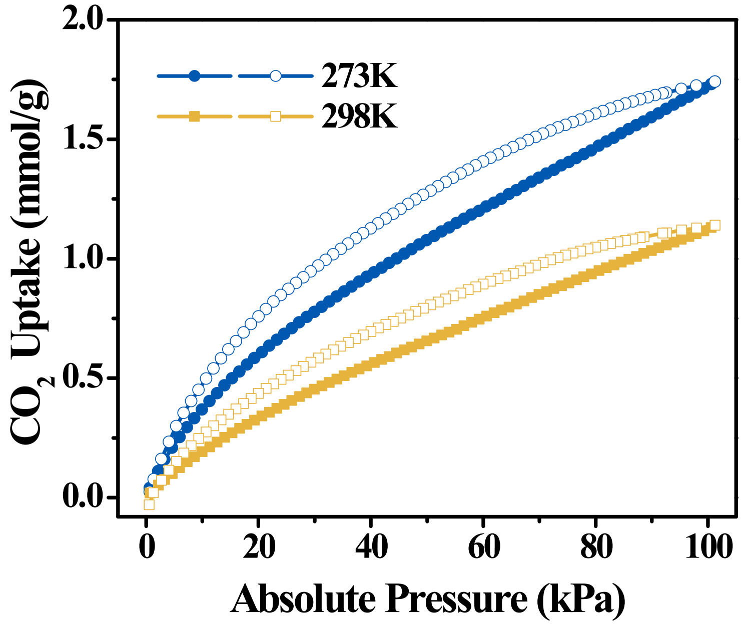

Fig. S9 CO_2 adsorption isotherms for PI-COF-2 at 273K and 298K, respectively.

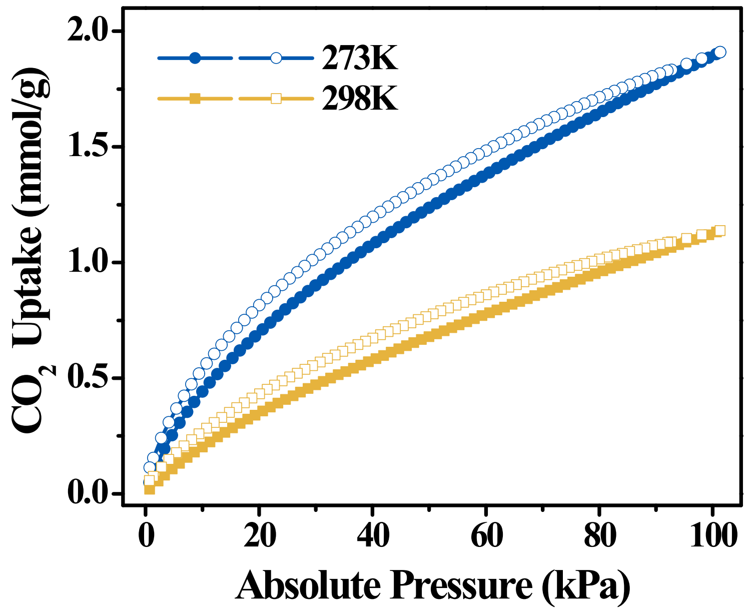

Fig. S10 CO_2 adsorption isotherms for PI-COF-TT at 273K and 298K, respectively.

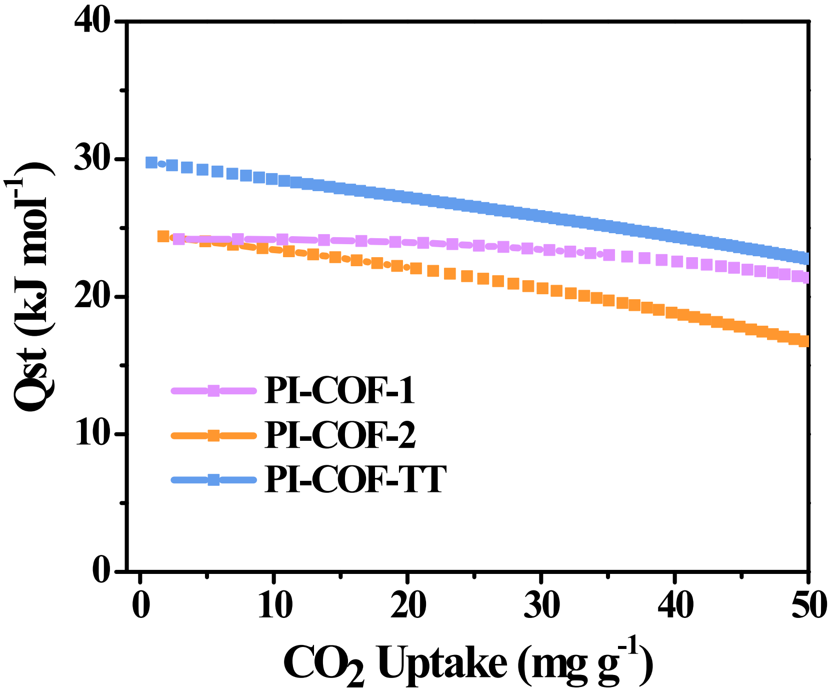

Fig. S11 Q_{st} for CO_2 adsorption of PI-COFs.

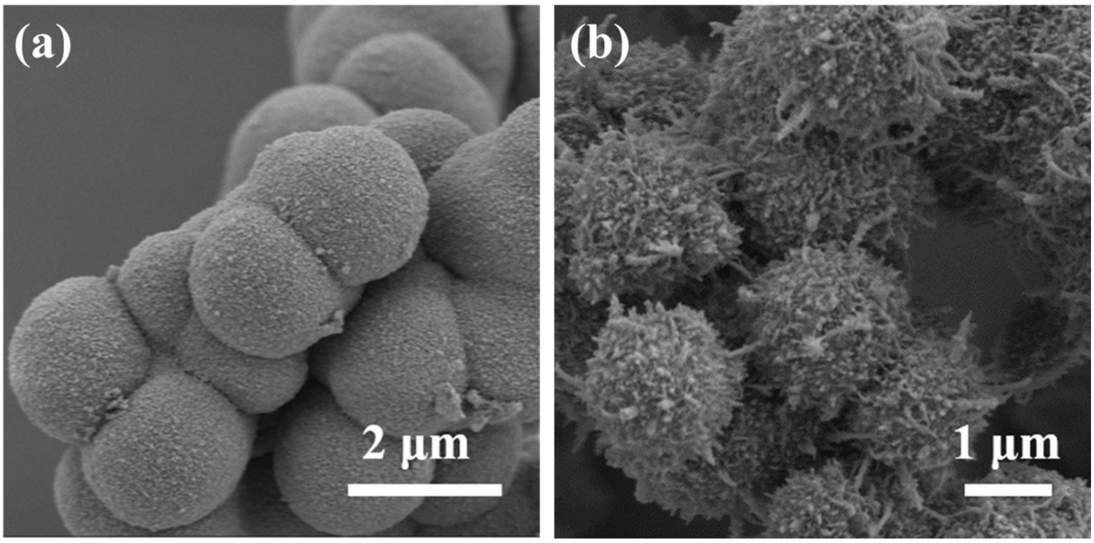

Fig. S12 SEM image of PI-COF-1 and PI-COF-2.

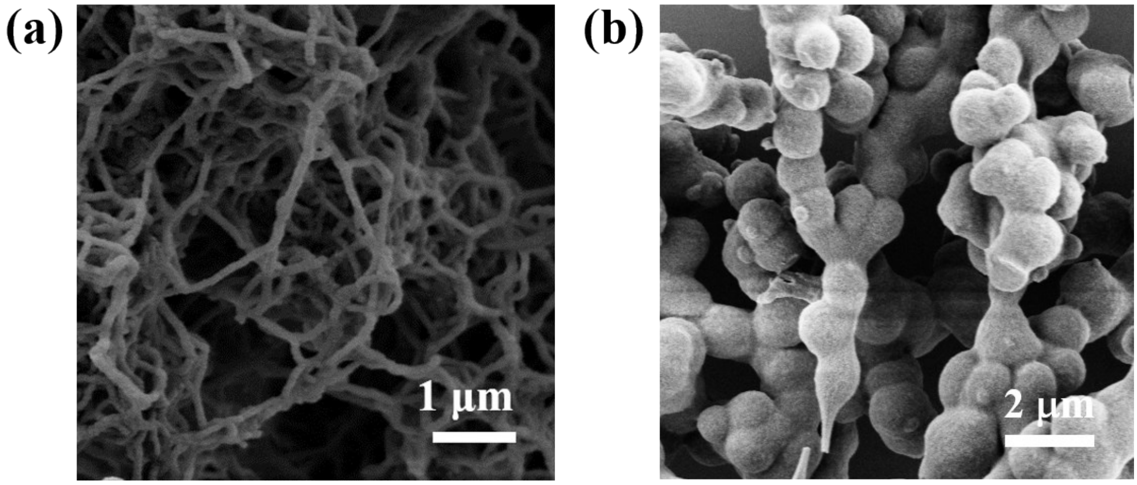

Fig. S13 SEM image of fresh PI-COF-TT.

A "sphere-like" morphology of PI-COF-TT can also be obtained as previously reported by the increasing the crystal intensity of pyromellitic dianhydride. The different morphology was probably ascribed to the concentration of pyromellitic dianhydride in the reaction solution. With the increment of the crystal intensity of pyromellitic dianhydride, the concentration of pyromellitic dianhydride in the reaction solution reduced, resulting in the formation of "sphere-like" morphology.

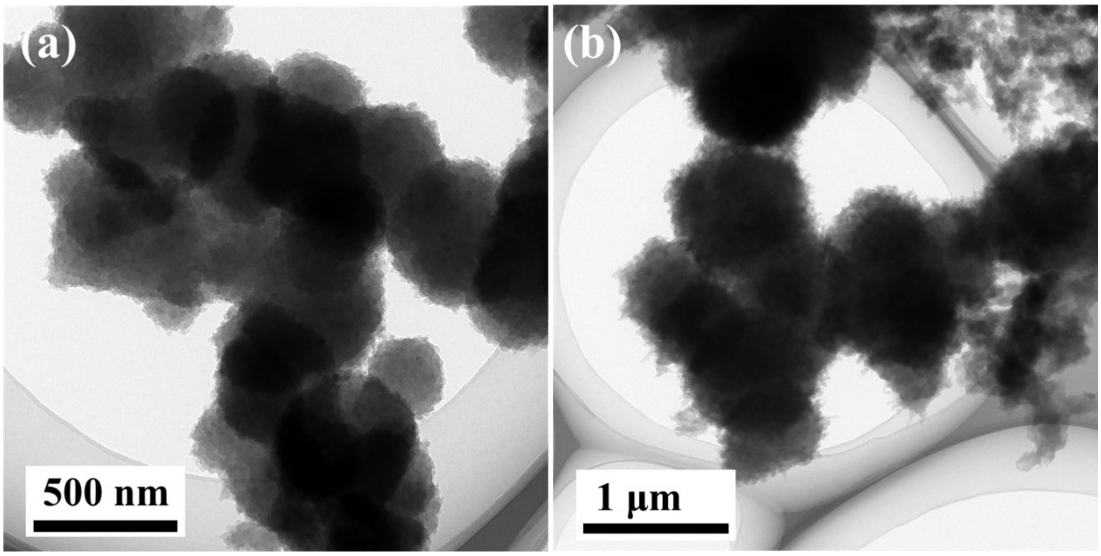

Fig. S14 TEM images for PI-COF-1 (a) and PI-COF-2 (b).

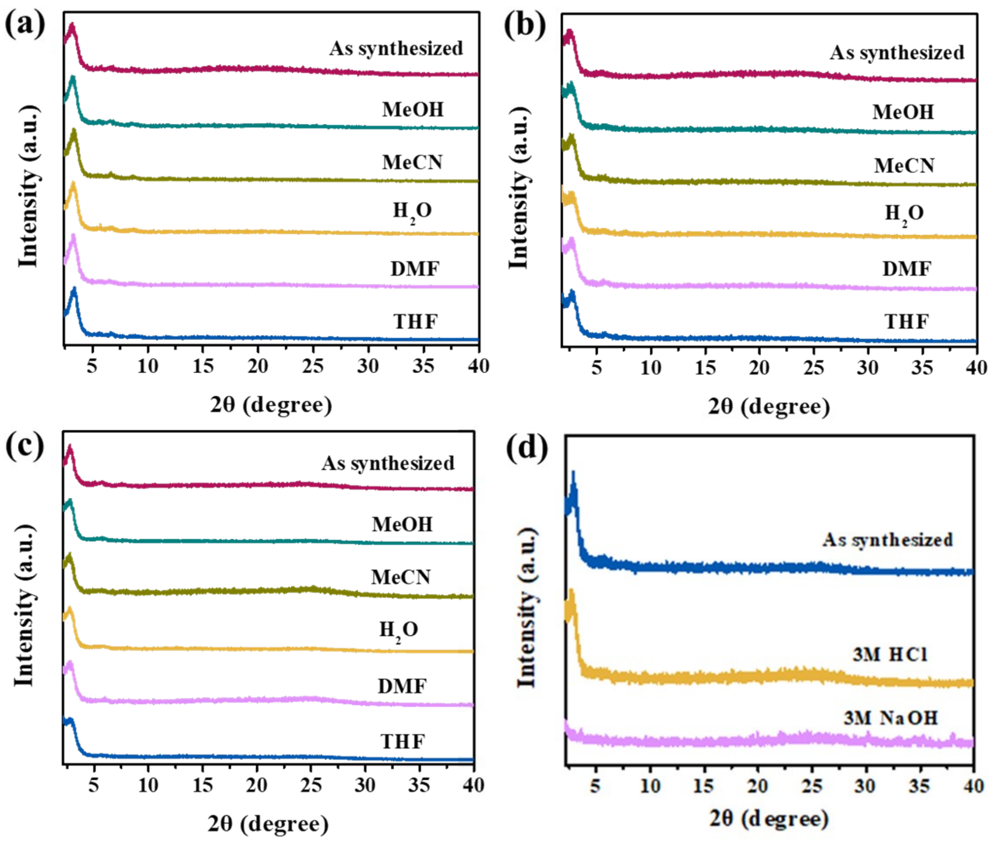

Fig. S15 PXRD patterns of PI-COF-1 (a), PI-COF-2 (b) and PI-COF-TT (c) treated for 1 day in MeOH, MeCN, H_2O, DMF and THF, respectively. PXRD patterns of PI-COF-TT (d) treated for 1 day in HCl, TEOA and NaOH aqueous solutions, respectively.

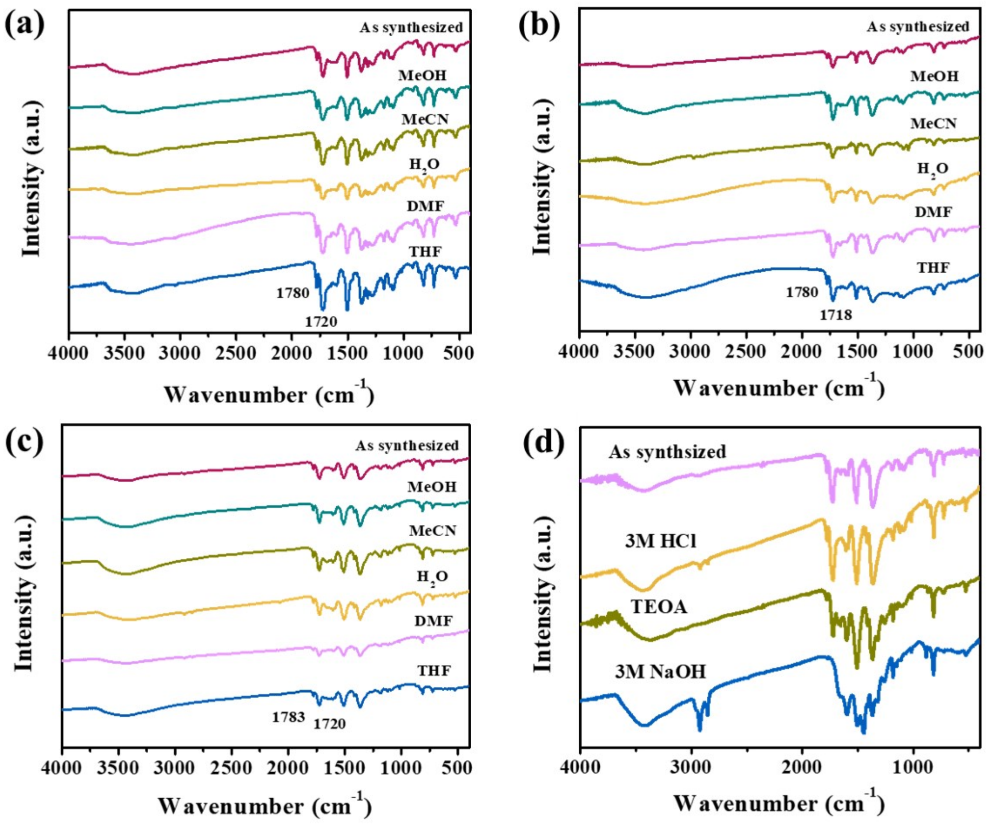

Fig. S16 FT-IR spectra of PI-COF-1 (a), PI-COF-2 (b) and PI-COF-TT (c) treated for 1 day in MeOH, MeCN, H_2O, DMF and THF, respectively. FT-IR spectra of PI-COF-TT (d) treated for 1 day in HCl, TEOA and NaOH aqueous solutions, respectively.

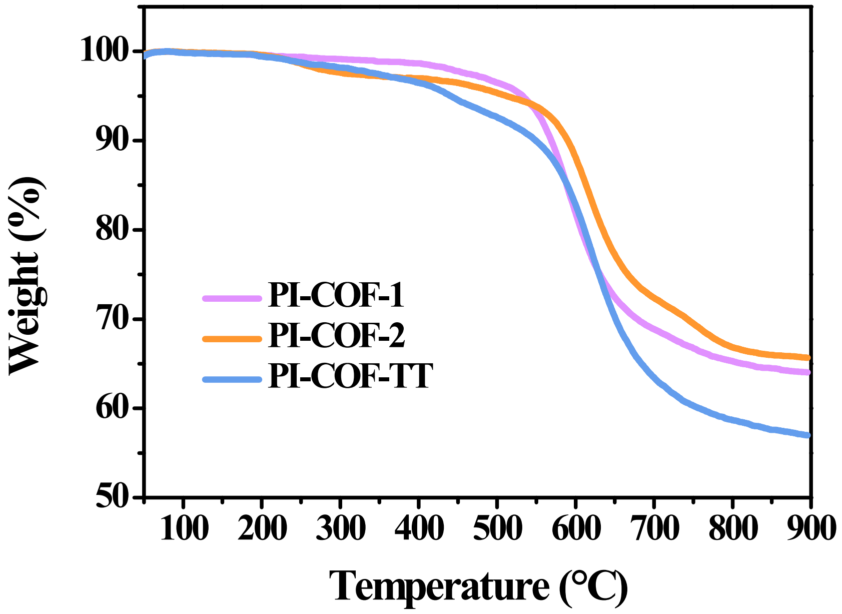

Fig. S17 TGA of PI-COFs.

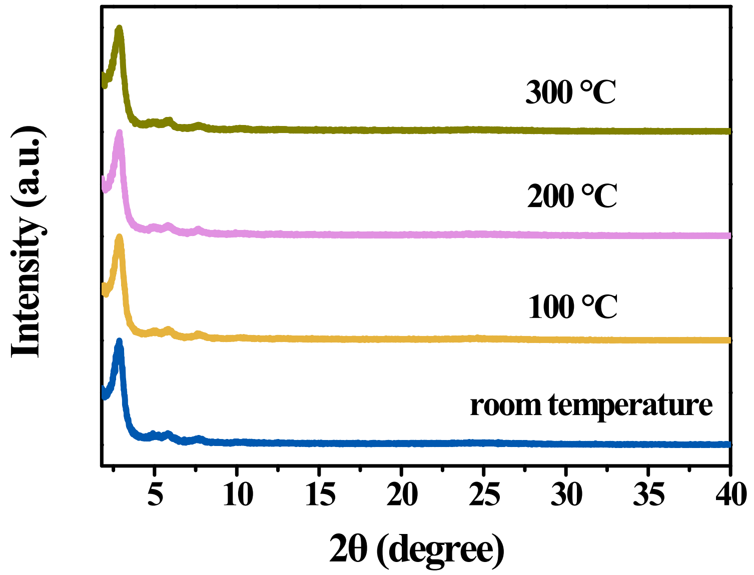

Fig. S18 High temperature XRD pattern of PI-COF-TT.

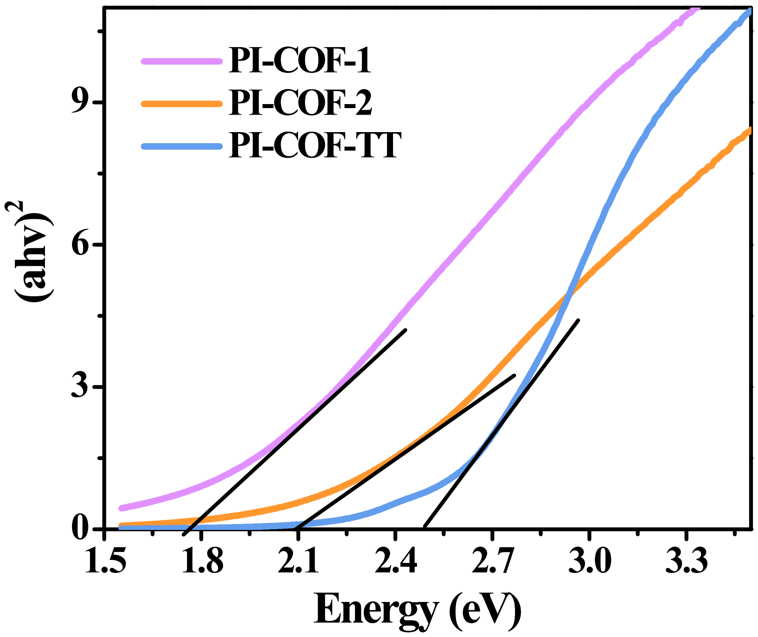

Fig. S19 The UV/vis absorption spectra and band gap of PI-COFs.

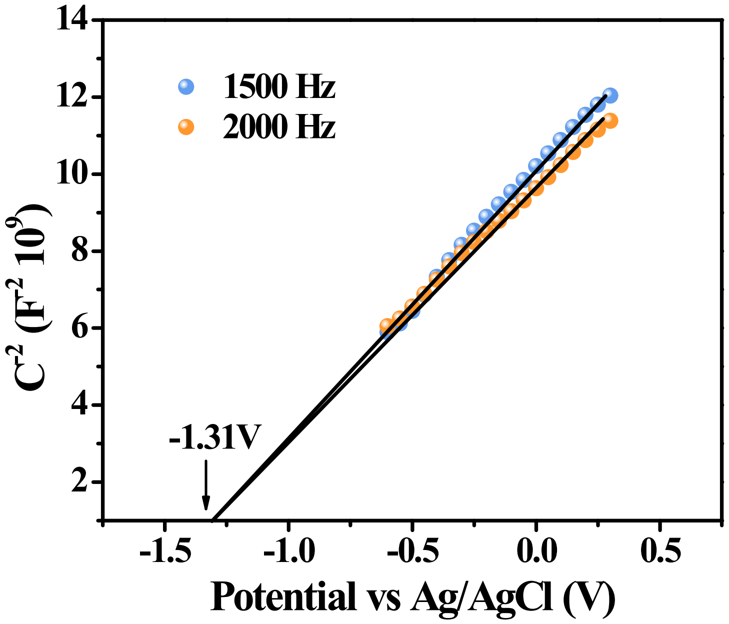

Fig. S20 Mott-Schottky plots of PI-COF-1.

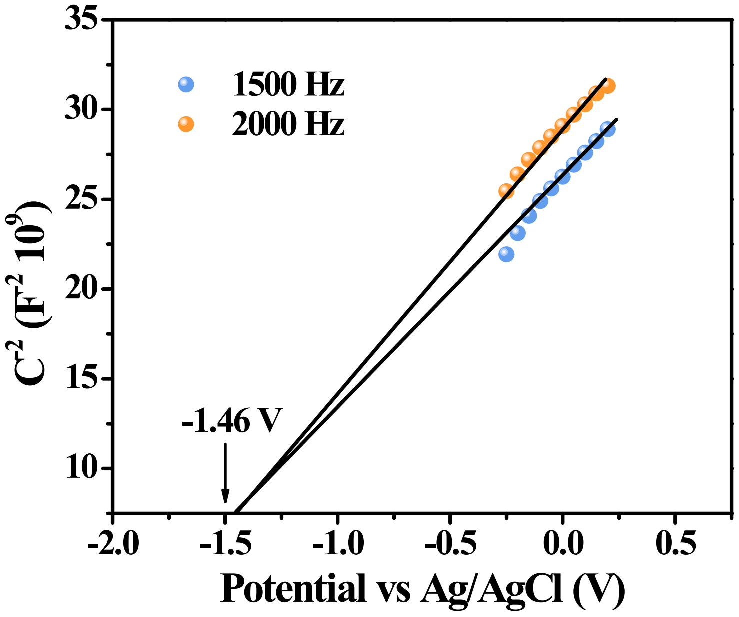

Fig. S21 Mott-Schottky plots of PI-COF-2.

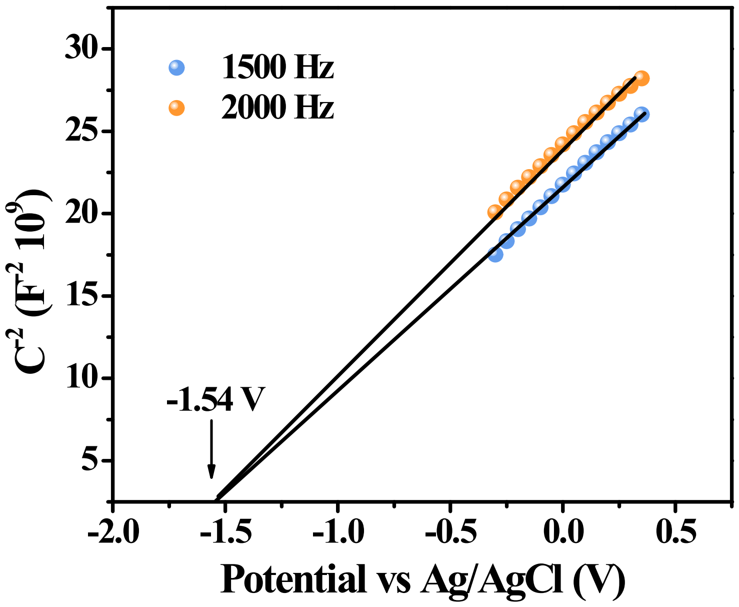

Fig. S22 Mott-Schottky plots of PI-COF-TT.

The flat band potential of PI-COF-1, PI-COF-2 and PI-COF-TT are located at -1.31 eV, -1.46 eV and -1.54 eV, respectively. The Mott-Schottky curves indicate typical n-type semiconductor behavior for all PI-COFs.

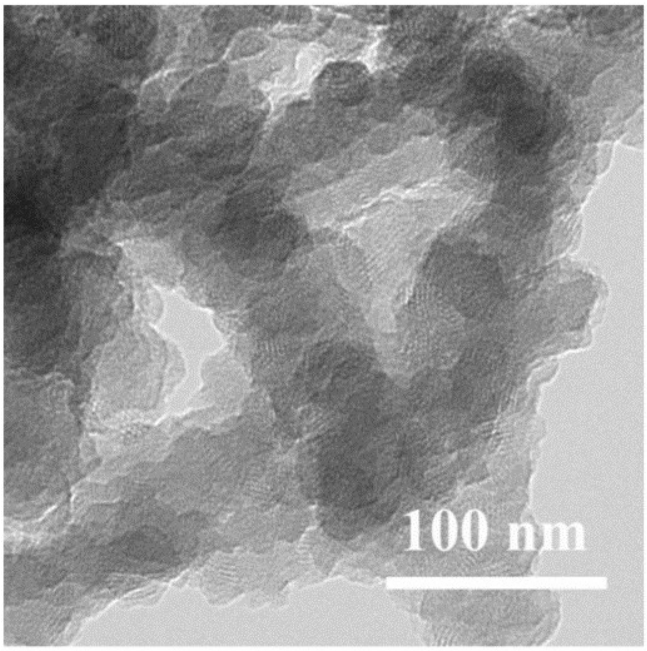

Fig. S23 TEM images of Ni@PI-COF-TT.

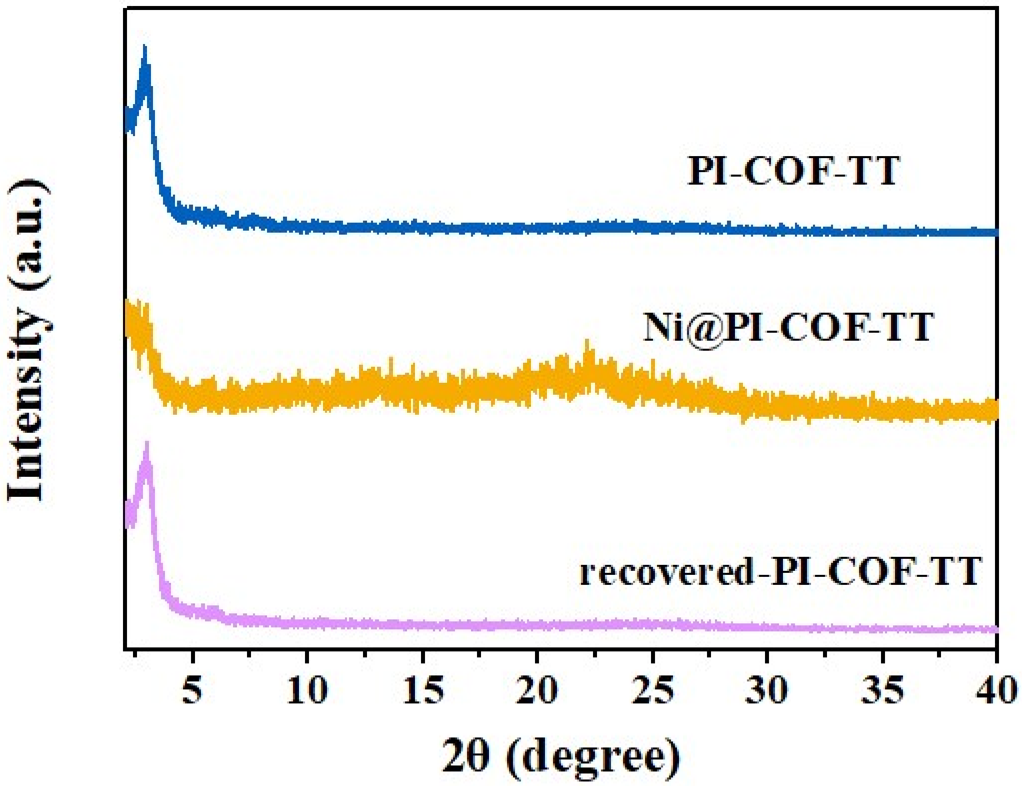

Fig. S24 PXRD patterns of PI-COF-TT, Ni@PI-COF-TT and PI-COF-TT after removal of $[Ni(bpy)_3]^{2+}$.

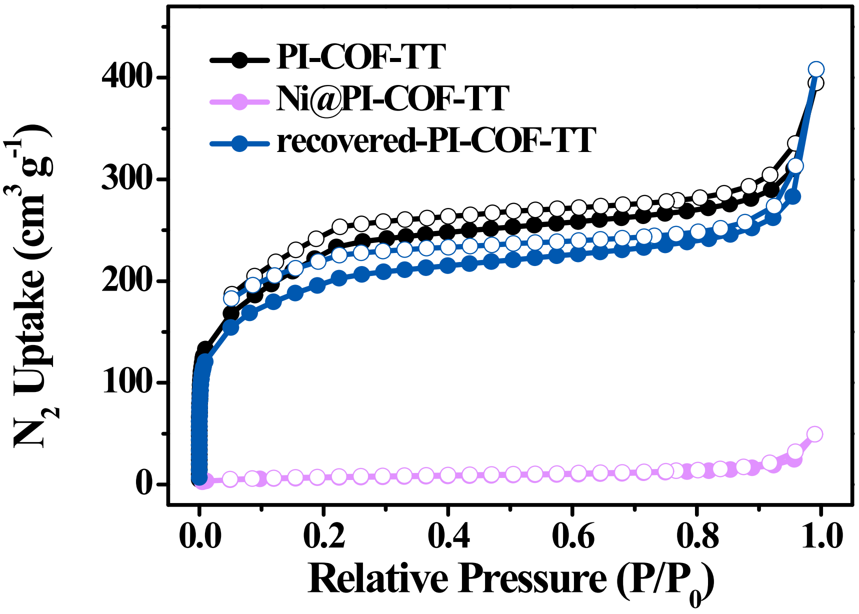

Fig. S25 The N_2 sorption isotherms for PI-COF-TT and Ni@PI-COF-TT.

The PXRD patterns and N_2 sorption isotherms showed the formation of $[Ni(bpy)_3]^{2+}$ complex was in the pore of PI-COF-TT.

Table S1. Photocatalytic reduction of CO_2 in aqueous solution for CO production.

Entries	Photocatalyst	Products (μmol h^{-1} g^{-1})	Select. of CO	Reaction solvent	Irradiation condition	Ref.
1	PI-COF-TT and $[Ni(bpy)_3]^{2+}$	CO (483)	93 %	MeCN/H_2O/TEOA (3:1:1)	300 W Xe lamp	This work
2	N_3–COF	CH_3OH (0.55)	99%	H_2O	λ > 420 (500W Xe lamp)	9
3	TTCOF-Zn	CO (2.06)	68%	H_2O	λ > 420 (300 W Xe lamp)	10
4	Re-COF	CO (625)	98%	MeCN/TEOA (3:0.2)	λ > 420 (225 W Xe lamp)	11
5	DA-CTF-Co	CO (155)	69%	MeCN/TEOA (2:1)	λ > 420 (225 W Xe lamp)	12
6	$ZnIn_2S_4$	CO (33.2)	71%	H_2O	300W Xe lamp with a standard AM 1.5 filter	13
7	Cu_2O/WO_3-001	CO (5.73)	65%	H_2O	λ > 400 (300 W Xe lamp)	14
8	BiOBr	CO (87.4)	70%	H_2O	λ > 400 (300 W Xe lamp)	15

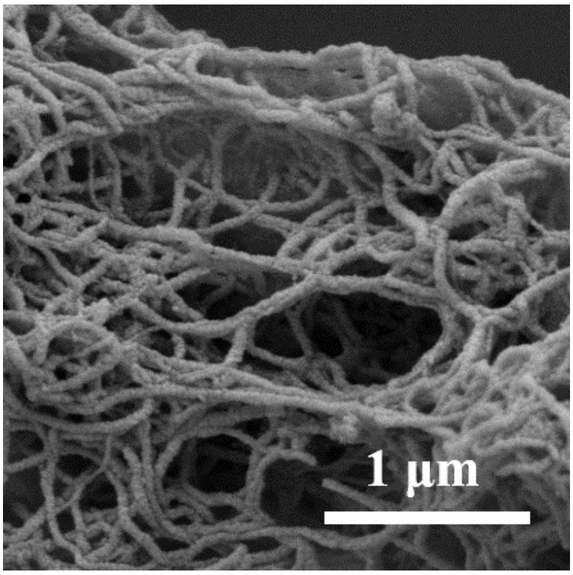

Fig. S26 SEM images of the recovered PI-COF-TT in CO_2 reduction reaction.

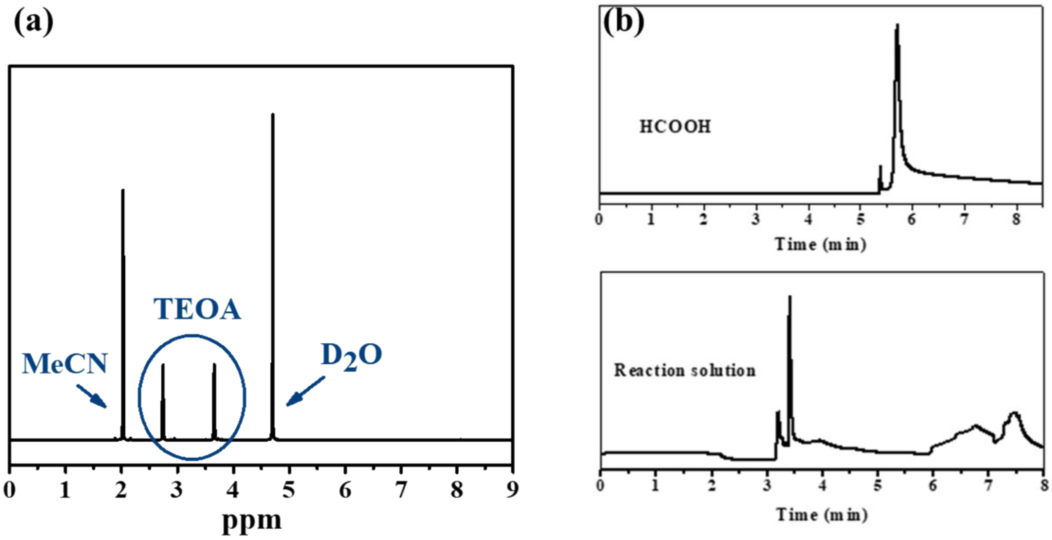

Fig. S27 ^{1}H NMR spectra (a) and HPLC (b) of the liquid phase taken from the reaction system after light irradiation for 4 h.

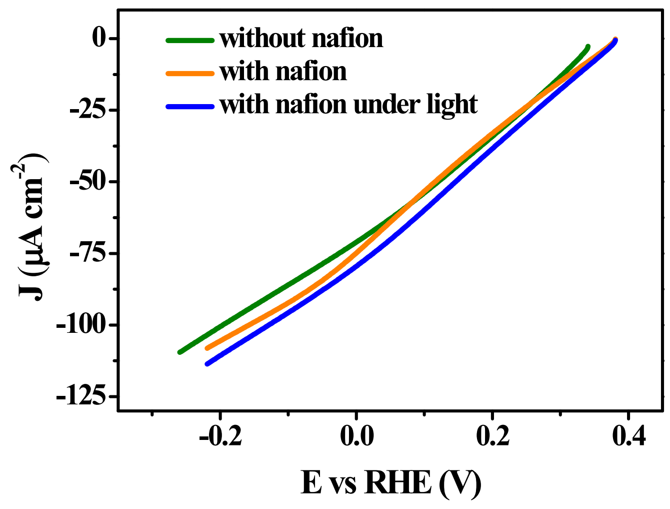

Fig. S28 Linear sweep voltammograms of electrodes coated with and without nafion in dark or under illumination.

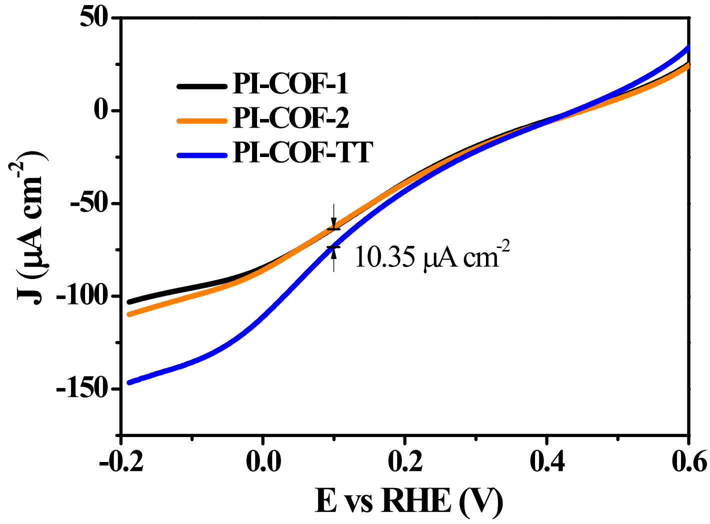

Fig. S29 Linear sweep voltammograms of electrodes coated with PI-COF-1, PI-COF-2 and PI-COF-TT.

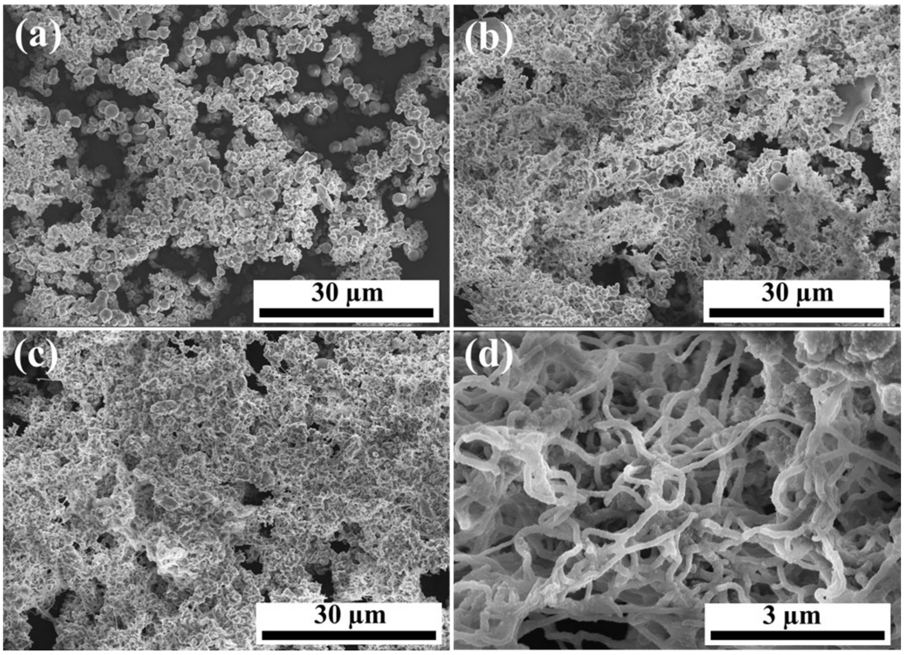

Fig. S30 SEM images of FTO glass electrodes of PI-COF-1 (a), PI-COF-2 (b) and PI-COF-TT (c, d)

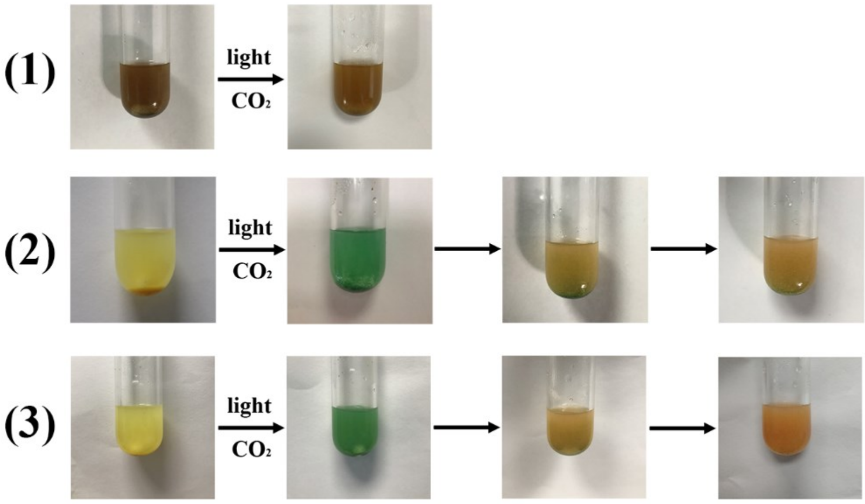

Fig. S31 Photos of the CO_2 photocatalytic reaction in presence of PI-COF-1 (1), PI-COF-2 (2) and PI-COF-TT (3), respectively.

Table S2. The color change of PI-COF-TT in acetonitrile solution under light irradiation in N_2 conditions.

Entry	Catalyst	Color change [a)]
1	PI-COF-TT	n.d.

2	PI-COF-TT/TEOA	yellow to green
3	PI-COF-TT/2,2'-bipyridyl	n.d.
4	PI-COF-TT/TEOA/2,2'-bipyridyl	yellow to green
5	PI-COF-TT/TEOA/2,2'-bipyridyl /$Ni(ClO_4)_2{\cdot}6H_2O$	yellow to green
6	2,2'-bipyridyl /TEOA	n.d.
7	2,2'-bipyridyl/TEOA/$Ni(ClO_4)_2{\cdot}6H_2O$	n.d.

[a] n.d. is not determined.

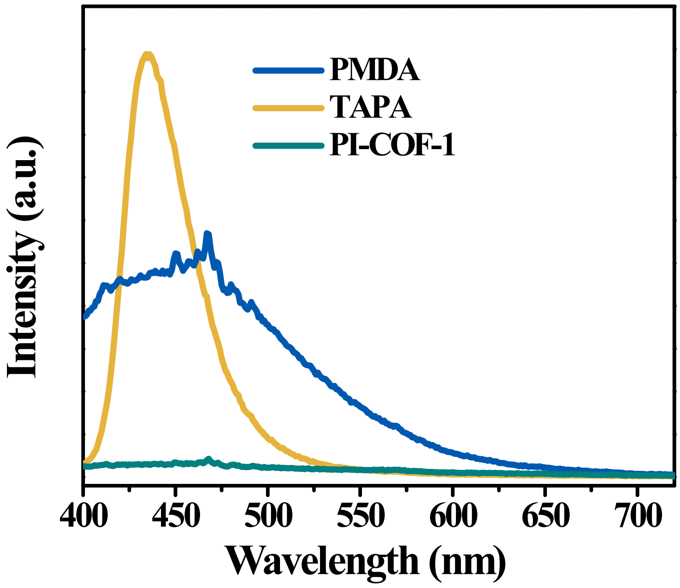

Fig. S32 Photoluminescence spectra (λ_{ex} =365 nm) of PI-COF-1 (solid sample).

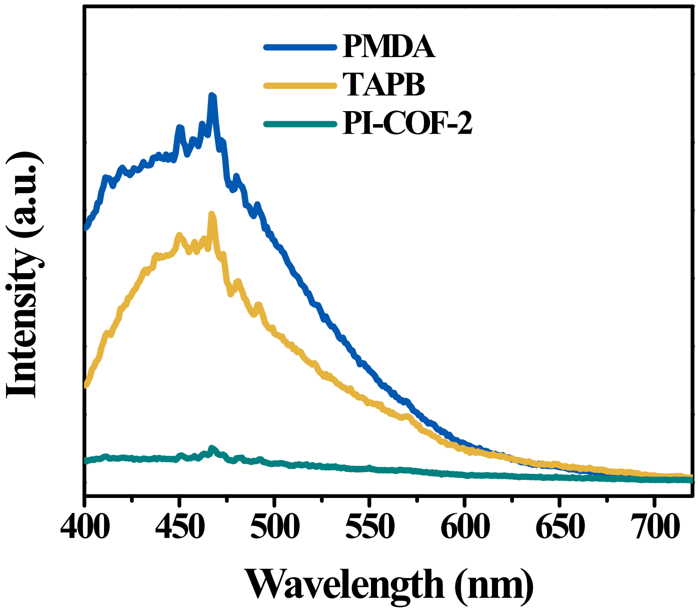

Fig. S33 Photoluminescence spectra (λ_{ex} =365 nm) of PI-COF-2 (solid sample).

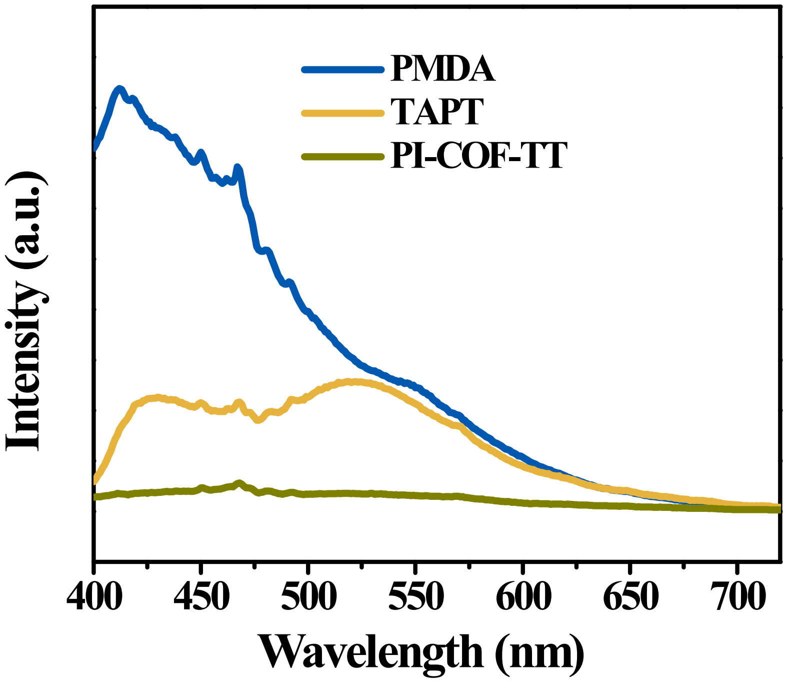

Fig. S34 Photoluminescence spectra (λ_{ex} =365 nm) of PI-COF-TT (solid sample).

The weak emission of PI-COFs results in impossible quench experiments by molecular Ni and/or TEOA.

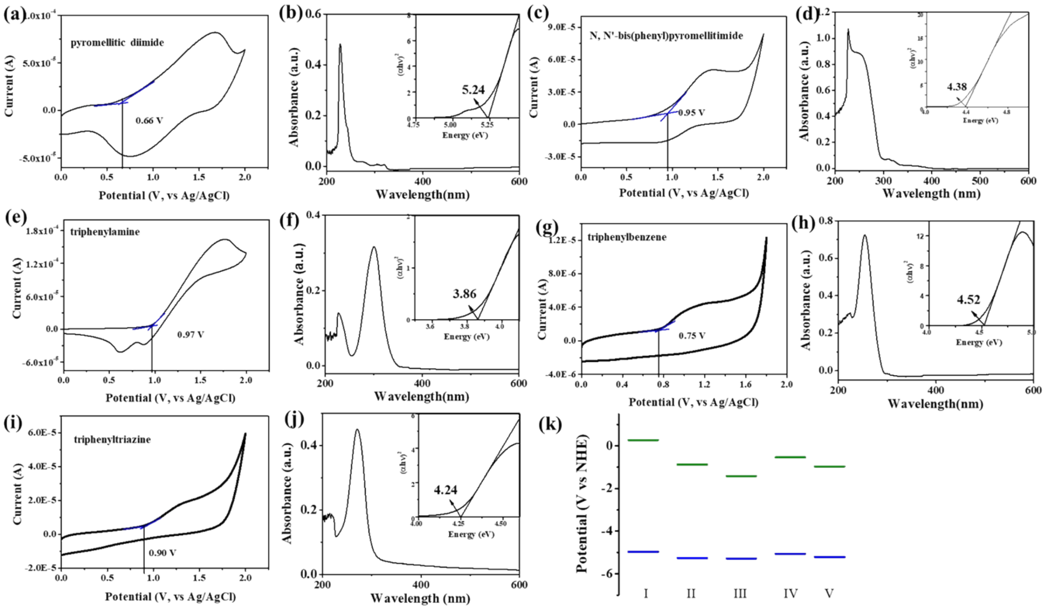

Fig. S35 Cyclic voltammograms and UV (inset Tauc plot) of pyromellitic diimide (a, b), N, N'-bis(phenyl)pyromellitimide (c, d), triphenylamine (e, f), triphenylbenzene (g, h), triphenyltriazine (i, j). (k) LUMO and HOMO levels of pyromellitic diimide (I), N, N'-bis(phenyl)pyromellitimide (II), triphenylamine (III), triphenylbenzene (IV), triphenyltriazine (V). The HOMO levels are estimated from the onset of the first oxidation waves from CV tests. The band gaps (Eg) were calculated from Tauc plot of UV. The relative positions of LUMO and HOMO are obtained according to the formula (HOMO = -[(eE^{OX} -eE(Fc/Fc^+) + 4.8 V)] eV, LUMO = HOMO+Eg, eE(Fc/Fc^+)=0.49 eV).

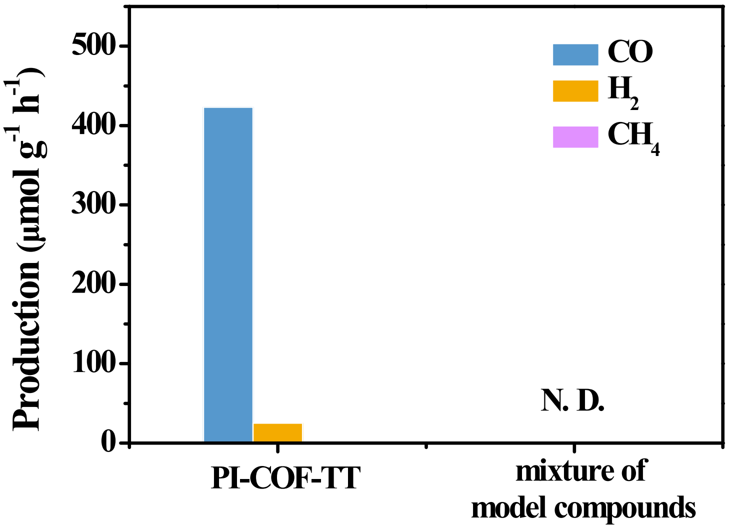

Fig. S36 Control experiments by using a physical mixture of pyromellitic diimide and triphenyltriazine as model compounds of building blocks in photoreduction of CO_2.

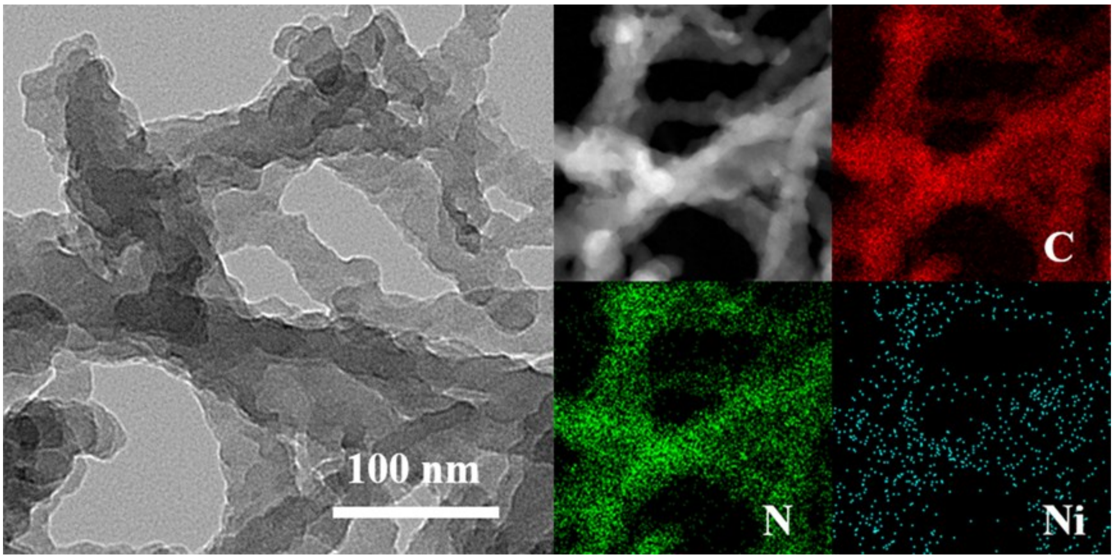

Fig. S37 TEM images and EDX elemental mapping of C, N and Ni for recovered PI-COF-TT from the photoreduction of CO_2 reaction without 2,2'-bipyridyl.

TEM images showed that no Ni clusters were observed on PI-COF-TT. EDX elemental mapping of the recovered PI-COF-TT with 2,2'-bipyridyl in the catalytic reactions showed negligible Ni signals than that from the reaction without 2,2'-

bipyridyl, suggesting the weak interactions between molecular Ni active sites and the COF.

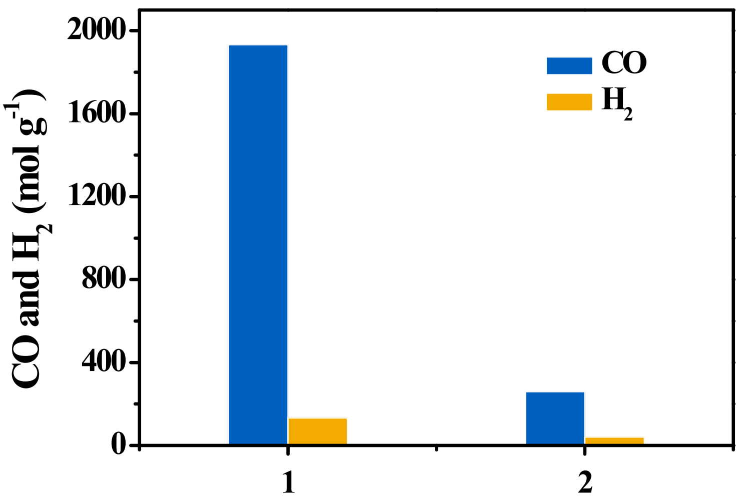

Fig. S38 Catalytic performance of PI-COF-TT with in-situ formed $[Ni(bpy)_3]^{2+}$ (1) and with direct impregnation of $[Ni(bpy)_3]^{2+}$ (2), respectively.

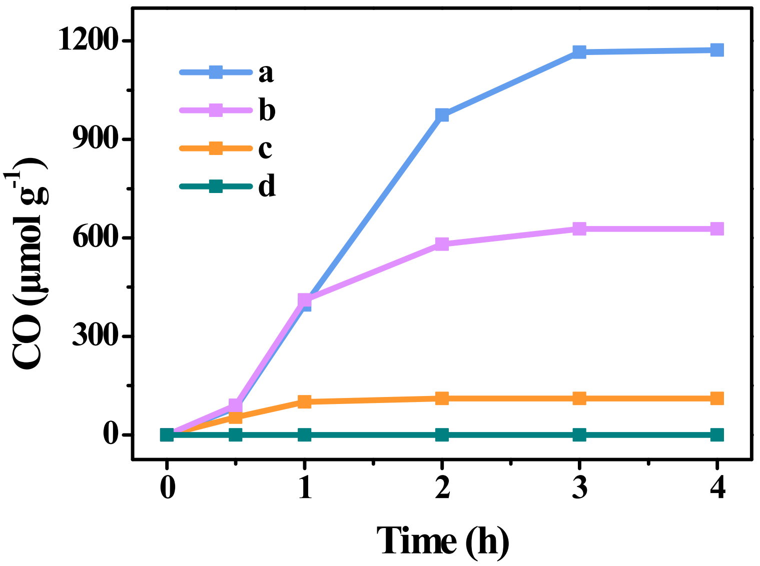

Fig. S39 The CO production over PI-COF-TT initiated by different light source. a: 2 h full light + 2 h visible light; b: 1 h full light + 3 h visible light; c: 0.5 h full light + 3.5 h visible light; d: 4 h visible light. Reaction conditions: PI-COF-TT (10mg), $Ni(ClO_4)_2{\cdot}6H_2O$ (1 mg, 2.7 μmol), 2,2'-bipyridyl (15 mg, 0.1 mmol), solvent (5 mL, acetonitrile/water/TEOA =3:1:1), CO_2 (1 atm).

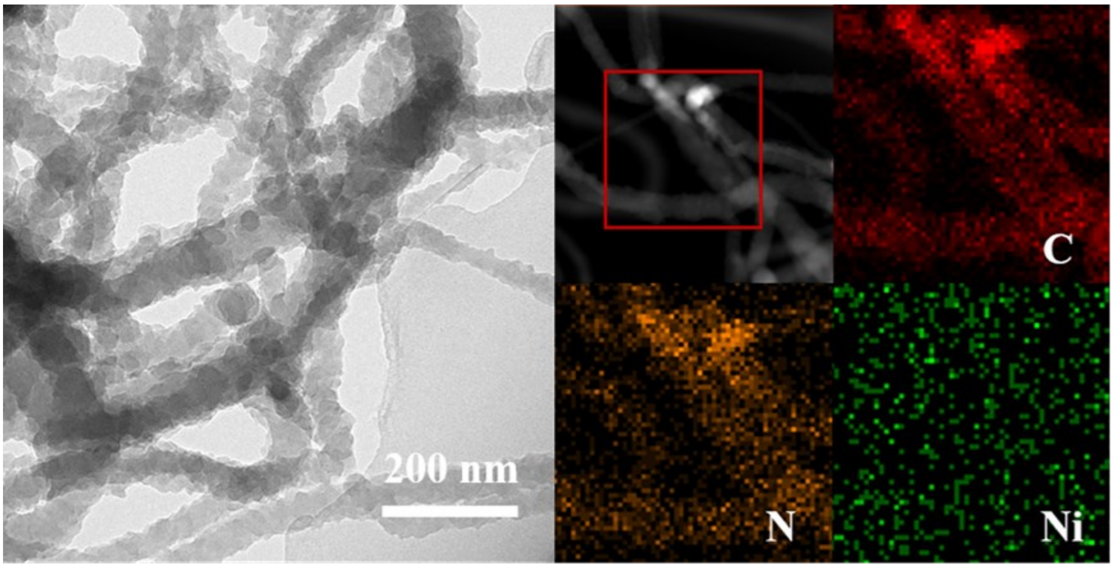

Fig. S40 TEM images and EDX elemental mapping of C, N and Ni for recovered PI-COF-TT from the photoreduction of CO_2 reaction with 2,2'-bipyridyl under visible light ($\lambda > 420$ nm).

TEM images showed that no Ni clusters were observed on the recovered PI-COF-TT from the photoreduction of CO_2 reaction with 2,2'-bipyridyl under visible light ($\lambda > 420$ nm). EDX elemental mapping of the recovered PI-COF-TT showed negligible Ni signals, suggesting the nearly no generation of $Ni(bpy)_2^0$ active sites under visible light.

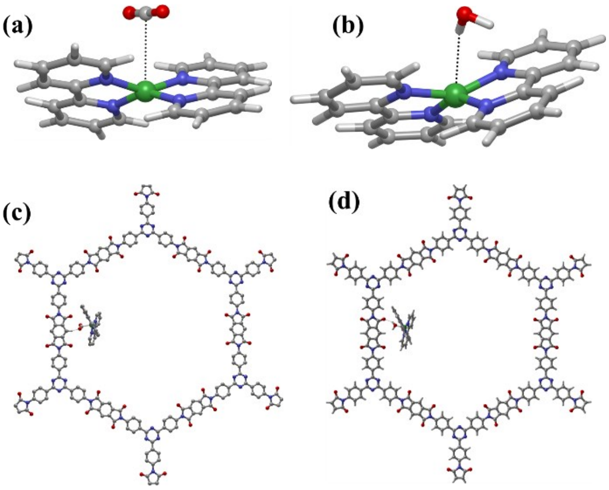

Fig. S41 Optimized structures of Ni-CO_2 (a), Ni-H_2O (b), Ni-COF-CO_2 (c) and Ni-COF-H_2O (d).

Table S3. Constrained the optimized structures of Ni-CO_2, Ni-H_2O, Ni-COF-CO_2 and Ni-COF-H_2O.

	Ni-CO_2	Ni-H_2O	Ni-COF-CO_2	Ni-COF-H_2O
ΔEabs(kcal/mol)	-7.7	-12.2	-101.5	-12.7
Ni-C (Å)[a)]	-3.09		2.24	
CO_2 angle (°)	175.3		155.8	

[a)] The distance of C in CO_2 with Ni atom in nickel complex.

Calculated Geometries Parameters of key optimized structures.

Geometrical coordinates (in fractional coordinate) of PI-COF-1 optimized by VASP

PI-COF-1: Space group: P1			
a=8.0352 Å b=31.6595 Å c=54.8359 Å			
α=90.0000° β=90.0000° γ=90.0000°			
**Atom**	**x**	**y**	**z**
C1	0.61293	0.53275	0.06632
C2	0.23711	0.53264	0.18753
C3	0.61293	0.54332	0.14228
C4	0.23711	0.54321	0.26349
C5	0.61293	0.42409	0.10959
C6	0.23711	0.42398	0.23079
C7	0.61356	0.53303	0.04087
C8	0.23774	0.53292	0.16208
C9	0.61356	0.58135	0.15515
C10	0.23774	0.58124	0.27635
C11	0.61356	0.38578	0.12217
C12	0.23774	0.38567	0.24338
C13	0.62288	0.46704	0.98694
C14	0.24706	0.46693	0.10815
C15	0.62288	0.69525	0.14912
C16	0.24706	0.69514	0.27032
C17	0.62288	0.33788	0.18213
C18	0.24706	0.33777	0.30334
C19	0.65071	0.48007	0.96096
C20	0.27489	0.47996	0.08217
C21	0.65071	0.7277	0.16862
C22	0.27489	0.72759	0.28983
C23	0.65071	0.2924	0.18861
C24	0.27489	0.29228	0.30981
C25	0.68791	0.53937	0.11917
C26	0.31209	0.53926	0.24038
C27	0.68791	0.61738	0.14517
C28	0.31209	0.61727	0.26638
C29	0.76461	0.54135	0.9394
C30	0.76461	0.72941	0.21004
C31	0.38879	0.7293	0.33125
O32	0.82141	0.56497	0.99442
O33	0.44558	0.56486	0.11563
O34	0.82141	0.63506	0.19434

O35	0.44558	0.63495	0.31555
O36	0.82141	0.30013	0.12943
O37	0.44558	0.30002	0.25063
H38	0.55203	0.55877	0.07574
H39	0.17621	0.55866	0.19694
H40	0.55203	0.51619	0.15058
H41	0.17621	0.51608	0.27179
H42	0.55203	0.42521	0.09187
H43	0.17621	0.4251	0.21308
H44	0.55158	0.55865	0.03115
H45	0.17576	0.55854	0.15236
H46	0.55158	0.58313	0.17282
H47	0.17576	0.58302	0.29402
H48	0.55158	0.35839	0.11422
H49	0.17576	0.35828	0.23543
H50	0.82168	0.57264	0.9394
H51	0.82168	0.71377	0.22569
H52	0.44586	0.71365	0.34689
N53	0.68791	0.65672	0.15828
N54	0.31209	0.65661	0.27949
C55	0.61293	0.03275	0.56632
C56	0.23711	0.03264	0.68753
C57	0.61293	0.04332	0.64228
C58	0.23711	0.04321	0.76349
C59	0.61293	0.92409	0.60959
C60	0.23711	0.92398	0.73079
C61	0.61356	0.03303	0.54087
C62	0.23774	0.03292	0.66208
C63	0.61356	0.08135	0.65515
C64	0.23774	0.08124	0.77635
C65	0.61356	0.88578	0.62217
C66	0.23774	0.88567	0.74338
C67	0.62288	0.96704	0.48694
C68	0.24706	0.96693	0.60815
C69	0.62288	0.19525	0.64912
C70	0.24706	0.19514	0.77032
C71	0.62288	0.83788	0.68213
C72	0.24706	0.83777	0.80334
C73	0.65071	0.98007	0.46096
C74	0.27489	0.97996	0.58217
C75	0.65071	0.2277	0.66862
C76	0.27489	0.22759	0.78983
C77	0.65071	0.7924	0.68861

C78	0.27489	0.79228	0.80981
C79	0.68791	0.03937	0.61917
C80	0.31209	0.03926	0.74038
C81	0.68791	0.11738	0.64517
C82	0.31209	0.11727	0.76638
C83	0.76461	0.04135	0.4394
C84	0.76461	0.22941	0.71004
C85	0.38879	0.2293	0.83125
O86	0.82141	0.06497	0.49442
O87	0.44558	0.06486	0.61563
O88	0.82141	0.13506	0.69434
O89	0.44558	0.13495	0.81555
O90	0.82141	0.80013	0.62943
O91	0.44558	0.80002	0.75063
H92	0.55203	0.05877	0.57574
H93	0.17621	0.05866	0.69694
H94	0.55203	0.01619	0.65058
H95	0.17621	0.01608	0.77179
H96	0.55203	0.92521	0.59187
H97	0.17621	0.9251	0.71308
H98	0.55158	0.05865	0.53115
H99	0.17576	0.05854	0.65236
H100	0.55158	0.08313	0.67282
H101	0.17576	0.08302	0.79402
H102	0.55158	0.85839	0.61422
H103	0.17576	0.85828	0.73543
H104	0.82168	0.07264	0.4394
H105	0.82168	0.21377	0.72569
H106	0.44586	0.21365	0.84689
N107	0.68791	0.15672	0.65828
N108	0.31209	0.15661	0.77949
C109	0.76289	0.46736	0.06632
C110	0.38707	0.46725	0.18753
C111	0.76289	0.45679	0.14228
C112	0.38707	0.45668	0.26349
C113	0.76289	0.57602	0.10959
C114	0.38707	0.57591	0.23079
C115	0.76226	0.46708	0.04087
C116	0.38644	0.46697	0.16208
C117	0.76226	0.41876	0.15515
C118	0.38644	0.41865	0.27635
C119	0.76226	0.61433	0.12217
C120	0.38644	0.61422	0.24338

C121	0.75294	0.53307	0.98694
C122	0.37712	0.53296	0.10815
C123	0.75294	0.30486	0.14912
C124	0.37712	0.30475	0.27032
C125	0.75294	0.66223	0.18213
C126	0.37712	0.66212	0.30334
C127	0.72511	0.52004	0.96096
C128	0.34929	0.51993	0.08217
C129	0.72511	0.27241	0.16862
C130	0.34929	0.2723	0.28983
C131	0.72511	0.70772	0.18861
C132	0.34929	0.7076	0.30981
C133	0.68791	0.46074	0.11917
C134	0.31209	0.46063	0.24038
C135	0.68791	0.38273	0.14517
C136	0.31209	0.38262	0.26638
C137	0.61121	0.45876	0.9394
C138	0.61121	0.2707	0.21004
C139	0.23539	0.27059	0.33125
O140	0.55442	0.43514	0.99442
O141	0.17859	0.43503	0.11563
O142	0.55442	0.36505	0.19434
O143	0.17859	0.36494	0.31555
O144	0.55442	0.69998	0.12943
O145	0.17859	0.69987	0.25063
H146	0.82379	0.44134	0.07574
H147	0.44797	0.44123	0.19694
H148	0.82379	0.48392	0.15058
H149	0.44797	0.48381	0.27179
H150	0.82379	0.5749	0.09187
H151	0.44797	0.57479	0.21308
H152	0.82424	0.44146	0.03115
H153	0.44842	0.44135	0.15236
H154	0.82424	0.41698	0.17282
H155	0.44842	0.41687	0.29402
H156	0.82424	0.64172	0.11422
H157	0.44842	0.64161	0.23543
H158	0.55414	0.42748	0.9394
H159	0.55414	0.28635	0.22569
H160	0.17832	0.28623	0.34689
N161	0.68791	0.34339	0.15828
N162	0.31209	0.34328	0.27949
C163	0.76289	0.96736	0.56632

C164	0.38707	0.96725	0.68753
C165	0.76289	0.95679	0.64228
C166	0.38707	0.95668	0.76349
C167	0.76289	0.07602	0.60959
C168	0.38707	0.07591	0.73079
C169	0.76226	0.96708	0.54087
C170	0.38644	0.96697	0.66208
C171	0.76226	0.91876	0.65515
C172	0.38644	0.91865	0.77635
C173	0.76226	0.11433	0.62217
C174	0.38644	0.11422	0.74338
C175	0.75294	0.03307	0.48694
C176	0.37712	0.03296	0.60815
C177	0.75294	0.80486	0.64912
C178	0.37712	0.80475	0.77032
C179	0.75294	0.16223	0.68213
C180	0.37712	0.16212	0.80334
C181	0.72511	0.02004	0.46096
C182	0.34929	0.01993	0.58217
C183	0.72511	0.77241	0.66862
C184	0.34929	0.7723	0.78983
C185	0.72511	0.20772	0.68861
C186	0.34929	0.2076	0.80981
C187	0.68791	0.96074	0.61917
C188	0.31209	0.96063	0.74038
C189	0.68791	0.88273	0.64517
C190	0.31209	0.88262	0.76638
C191	0.61121	0.95876	0.4394
C192	0.61121	0.7707	0.71004
C193	0.23539	0.77059	0.83125
O194	0.55442	0.93514	0.49442
O195	0.17859	0.93503	0.61563
O196	0.55442	0.86505	0.69434
O197	0.17859	0.86494	0.81555
O198	0.55442	0.19998	0.62943
O199	0.17859	0.19987	0.75063
H200	0.82379	0.94134	0.57574
H201	0.44797	0.94123	0.69694
H202	0.82379	0.98392	0.65058
H203	0.44797	0.98381	0.77179
H204	0.82379	0.0749	0.59187
H205	0.44797	0.07479	0.71308
H206	0.82424	0.94146	0.53115

H207	0.44842	0.94135	0.65236
H208	0.82424	0.91698	0.67282
H209	0.44842	0.91687	0.79402
H210	0.82424	0.14172	0.61422
H211	0.44842	0.14161	0.73543
H212	0.55414	0.92748	0.4394
H213	0.55414	0.78635	0.72569
H214	0.17832	0.78623	0.84689
N215	0.68791	0.84339	0.65828
N216	0.31209	0.84328	0.77949
C217	0.38707	0.46725	0.93368
C218	0.76289	0.46736	0.81247
C219	0.38707	0.45668	0.85772
C220	0.76289	0.45679	0.73651
C221	0.38707	0.57591	0.89041
C222	0.76289	0.57602	0.76921
C223	0.38644	0.46697	0.95913
C224	0.76226	0.46708	0.83792
C225	0.38644	0.41865	0.84485
C226	0.76226	0.41876	0.72365
C227	0.38644	0.61422	0.87783
C228	0.76226	0.61433	0.75662
C229	0.37712	0.53296	0.01306
C230	0.75294	0.53307	0.89185
C231	0.37712	0.30475	0.85088
C232	0.75294	0.30486	0.72968
C233	0.37712	0.66212	0.81787
C234	0.75294	0.66223	0.69666
C235	0.34929	0.51993	0.03904
C236	0.72511	0.52004	0.91783
C237	0.34929	0.2723	0.83138
C238	0.72511	0.27241	0.71017
C239	0.34929	0.7076	0.81139
C240	0.72511	0.70772	0.69019
C241	0.31209	0.46063	0.88083
C242	0.68791	0.46074	0.75962
C243	0.31209	0.38262	0.85483
C244	0.68791	0.38273	0.73362
C245	0.23539	0.45865	0.0606
C246	0.23539	0.27059	0.78996
C247	0.61121	0.2707	0.66875
O248	0.17859	0.43503	0.00558
O249	0.55442	0.43514	0.88437

O250	0.17859	0.36494	0.80566
O251	0.55442	0.36505	0.68445
O252	0.17859	0.69987	0.87057
O253	0.55442	0.69998	0.74937
H254	0.44797	0.44123	0.92426
H255	0.82379	0.44134	0.80306
H256	0.44797	0.48381	0.84942
H257	0.82379	0.48392	0.72821
H258	0.44797	0.57479	0.90813
H259	0.82379	0.5749	0.78692
H260	0.44842	0.44135	0.96885
H261	0.82424	0.44146	0.84764
H262	0.44842	0.41687	0.82718
H263	0.82424	0.41698	0.70598
H264	0.44842	0.64161	0.88578
H265	0.82424	0.64172	0.76457
H266	0.17832	0.42736	0.0606
H267	0.17832	0.28623	0.77431
H268	0.55414	0.28635	0.65311
N269	0.31209	0.34328	0.84172
N270	0.68791	0.34339	0.72051
C271	0.38707	0.96725	0.43368
C272	0.76289	0.96736	0.31247
C273	0.38707	0.95668	0.35772
C274	0.76289	0.95679	0.23651
C275	0.38707	0.07591	0.39041
C276	0.76289	0.07602	0.26921
C277	0.38644	0.96697	0.45913
C278	0.76226	0.96708	0.33792
C279	0.38644	0.91865	0.34485
C280	0.76226	0.91876	0.22365
C281	0.38644	0.11422	0.37783
C282	0.76226	0.11433	0.25662
C283	0.37712	0.03296	0.51306
C284	0.75294	0.03307	0.39185
C285	0.37712	0.80475	0.35088
C286	0.75294	0.80486	0.22968
C287	0.37712	0.16212	0.31787
C288	0.75294	0.16223	0.19666
C289	0.34929	0.01993	0.53904
C290	0.72511	0.02004	0.41783
C291	0.34929	0.7723	0.33138
C292	0.72511	0.77241	0.21017

C293	0.34929	0.2076	0.31139
C294	0.72511	0.20772	0.19019
C295	0.31209	0.96063	0.38083
C296	0.68791	0.96074	0.25962
C297	0.31209	0.88262	0.35483
C298	0.68791	0.88273	0.23362
C299	0.23539	0.95865	0.5606
C300	0.23539	0.77059	0.28996
C301	0.61121	0.7707	0.16875
O302	0.17859	0.93503	0.50558
O303	0.55442	0.93514	0.38437
O304	0.17859	0.86494	0.30566
O305	0.55442	0.86505	0.18445
O306	0.17859	0.19987	0.37057
O307	0.55442	0.19998	0.24937
H308	0.44797	0.94123	0.42426
H309	0.82379	0.94134	0.30306
H310	0.44797	0.98381	0.34942
H311	0.82379	0.98392	0.22821
H312	0.44797	0.07479	0.40813
H313	0.82379	0.0749	0.28692
H314	0.44842	0.94135	0.46885
H315	0.82424	0.94146	0.34764
H316	0.44842	0.91687	0.32718
H317	0.82424	0.91698	0.20598
H318	0.44842	0.14161	0.38578
H319	0.82424	0.14172	0.26457
H320	0.17832	0.92736	0.5606
H321	0.17832	0.78623	0.27431
H322	0.55414	0.78635	0.15311
N323	0.31209	0.84328	0.34172
N324	0.68791	0.84339	0.22051
C325	0.23711	0.53264	0.93368
C326	0.61293	0.53275	0.81247
C327	0.23711	0.54321	0.85772
C328	0.61293	0.54332	0.73651
C329	0.23711	0.42398	0.89041
C330	0.61293	0.42409	0.76921
C331	0.23774	0.53292	0.95913
C332	0.61356	0.53303	0.83792
C333	0.23774	0.58124	0.84485
C334	0.61356	0.58135	0.72365
C335	0.23774	0.38567	0.87783

C336	0.61356	0.38578	0.75662
C337	0.24706	0.46693	0.01306
C338	0.62288	0.46704	0.89185
C339	0.24706	0.69514	0.85088
C340	0.62288	0.69525	0.72968
C341	0.24706	0.33777	0.81787
C342	0.62288	0.33788	0.69666
C343	0.27489	0.47996	0.03904
C344	0.65071	0.48007	0.91783
C345	0.27489	0.72759	0.83138
C346	0.65071	0.7277	0.71017
C347	0.27489	0.29228	0.81139
C348	0.65071	0.2924	0.69019
C349	0.31209	0.53926	0.88083
C350	0.68791	0.53937	0.75962
C351	0.31209	0.61727	0.85483
C352	0.68791	0.61738	0.73362
C353	0.38879	0.54124	0.0606
C354	0.38879	0.7293	0.78996
C355	0.76461	0.72941	0.66875
O356	0.44558	0.56486	0.00558
O357	0.82141	0.56497	0.88437
O358	0.44558	0.63495	0.80566
O359	0.82141	0.63506	0.68445
O360	0.44558	0.30002	0.87057
O361	0.82141	0.30013	0.74937
H362	0.17621	0.55866	0.92426
H363	0.55203	0.55877	0.80306
H364	0.17621	0.51608	0.84942
H365	0.55203	0.51619	0.72821
H366	0.17621	0.4251	0.90813
H367	0.55203	0.42521	0.78692
H368	0.17576	0.55854	0.96885
H369	0.55158	0.55865	0.84764
H370	0.17576	0.58302	0.82718
H371	0.55158	0.58313	0.70598
H372	0.17576	0.35828	0.88578
H373	0.55158	0.35839	0.76457
H374	0.44586	0.57252	0.0606
H375	0.44586	0.71365	0.77431
H376	0.82168	0.71377	0.65311
N377	0.31209	0.65661	0.84172
N378	0.68791	0.65672	0.72051

C379	0.23711	0.03264	0.43368
C380	0.61293	0.03275	0.31247
C381	0.23711	0.04321	0.35772
C382	0.61293	0.04332	0.23651
C383	0.23711	0.92398	0.39041
C384	0.61293	0.92409	0.26921
C385	0.23774	0.03292	0.45913
C386	0.61356	0.03303	0.33792
C387	0.23774	0.08124	0.34485
C388	0.61356	0.08135	0.22365
C389	0.23774	0.88567	0.37783
C390	0.61356	0.88578	0.25662
C391	0.24706	0.96693	0.51306
C392	0.62288	0.96704	0.39185
C393	0.24706	0.19514	0.35088
C394	0.62288	0.19525	0.22968
C395	0.24706	0.83777	0.31787
C396	0.62288	0.83788	0.19666
C397	0.27489	0.97996	0.53904
C398	0.65071	0.98007	0.41783
C399	0.27489	0.22759	0.33138
C400	0.65071	0.2277	0.21017
C401	0.27489	0.79228	0.31139
C402	0.65071	0.7924	0.19019
C403	0.31209	0.03926	0.38083
C404	0.68791	0.03937	0.25962
C405	0.31209	0.11727	0.35483
C406	0.68791	0.11738	0.23362
C407	0.38879	0.04124	0.5606
C408	0.38879	0.2293	0.28996
C409	0.76461	0.22941	0.16875
O410	0.44558	0.06486	0.50558
O411	0.82141	0.06497	0.38437
O412	0.44558	0.13495	0.30566
O413	0.82141	0.13506	0.18445
O414	0.44558	0.80002	0.37057
O415	0.82141	0.80013	0.24937
H416	0.17621	0.05866	0.42426
H417	0.55203	0.05877	0.30306
H418	0.17621	0.01608	0.34942
H419	0.55203	0.01619	0.22821
H420	0.17621	0.9251	0.40813
H421	0.55203	0.92521	0.28692

H422	0.17576	0.05854	0.46885
H423	0.55158	0.05865	0.34764
H424	0.17576	0.08302	0.32718
H425	0.55158	0.08313	0.20598
H426	0.17576	0.85828	0.38578
H427	0.55158	0.85839	0.26457
H428	0.44586	0.07252	0.5606
H429	0.44586	0.21365	0.27431
H430	0.82168	0.21377	0.15311
N431	0.31209	0.15661	0.34172
N432	0.68791	0.15672	0.22051
C433	0.68791	0.50006	0.07985
C434	0.68791	0.50006	0.02785
N435	0.68791	0.50006	0.10606
N436	0.68791	0.50006	0.00162
C437	0.68791	0.00006	0.57985
C438	0.68791	0.00006	0.52785
N439	0.68791	0.00006	0.60606
N440	0.68791	0.00006	0.50162
C441	0.31209	0.49994	0.92015
C442	0.31209	0.49994	0.97215
N443	0.31209	0.49994	0.89394
N444	0.31209	0.49994	0.99838
C445	0.31209	-0.00006	0.42015
C446	0.31209	-0.00006	0.47215
N447	0.31209	-0.00006	0.39394
N448	0.31209	-0.00006	0.49838
C449	0.31209	0.49994	0.20106
C450	0.31209	0.49994	0.14906
N451	0.31209	0.49994	0.22727
N452	0.31209	0.49994	0.12283
C453	0.31209	-0.00006	0.70106
C454	0.31209	-0.00006	0.64906
N455	0.31209	-0.00006	0.72727
N456	0.31209	-0.00006	0.62283
C457	0.68791	0.50006	0.79894
C458	0.68791	0.50006	0.85094
N459	0.68791	0.50006	0.77273
N460	0.68791	0.50006	0.87717
C461	0.68791	0.00006	0.29894
C462	0.68791	0.00006	0.35094
N463	0.68791	0.00006	0.27273
N464	0.68791	0.00006	0.37717

□	□	□	□

Geometrical coordinates (in fractional coordinate) of PI-COF-2 optimized by VASP

PI-COF-2: Space group: P1			
a=7.2624 Å b=36.6977 Å c=63.5623 Å			
α=90° β=90° γ=90°			
**Atom**	**x**	**y**	**z**
C1	0.76689	0.59922	0.87555
C2	0.64347	0.39643	0.23612
C3	0.76696	0.46756	0.94668
C4	0.6434	0.52809	0.16499
C5	0.76701	0.42671	0.84529
C6	0.64335	0.56894	0.26638
C7	0.76732	0.63225	0.86473
C8	0.64304	0.3634	0.24694
C9	0.76734	0.46726	0.9686
C10	0.64302	0.52839	0.14307
C11	0.76736	0.39398	0.83417
C12	0.643	0.60167	0.2775
C13	0.76352	0.67163	0.81141
C14	0.64684	0.32402	0.30026
C15	0.76349	0.52755	0.01495
C16	0.64687	0.4681	0.09672
C17	0.76352	0.29431	0.84113
C18	0.64684	0.70135	0.27054
C19	0.73789	0.71102	0.80613
C20	0.67246	0.28463	0.30554
C21	0.73787	0.51578	0.03728
C22	0.67249	0.47987	0.07439
C23	0.73791	0.26669	0.82408
C24	0.67245	0.72896	0.28759
C25	0.70515	0.53063	0.90011
C26	0.70521	0.46502	0.21156
C27	0.70514	0.53138	0.87799
C28	0.70522	0.46427	0.23368
C29	0.70511	0.56659	0.86625

C30	0.70525	0.42906	0.24542
C31	0.70519	0.63363	0.84389
C32	0.70517	0.36202	0.26778
C33	0.63788	0.76639	0.8244
C34	0.77248	0.22926	0.28727
C35	0.63793	0.46069	0.05584
O36	0.58311	0.70618	0.85869
O37	0.82725	0.28947	0.25299
O38	0.58318	0.43934	0.0086
O39	0.82718	0.55631	0.10307
O40	0.58317	0.34793	0.80021
O41	0.82719	0.64773	0.31147
H42	0.82146	0.59897	0.89154
H43	0.5889	0.39668	0.22013
H44	0.82156	0.4437	0.93855
H45	0.5888	0.55195	0.17312
H46	0.8216	0.45082	0.83743
H47	0.58876	0.54483	0.27425
H48	0.81948	0.65677	0.87233
H49	0.59088	0.33888	0.23934
H50	0.81949	0.44359	0.97706
H51	0.59087	0.55206	0.13461
H52	0.81948	0.39314	0.81811
H53	0.59088	0.60252	0.29357
H54	0.70514	0.55636	0.90869
H55	0.70522	0.43929	0.20298
H56	0.58786	0.7804	0.83843
H57	0.8225	0.21525	0.27325
H58	0.58793	0.43264	0.05584
N59	0.7052	0.66744	0.83262
N60	0.70515	0.32821	0.27905
C61	0.76689	0.09922	0.37555
C62	0.64347	0.89643	0.73612
C63	0.76696	0.96756	0.44668
C64	0.6434	0.02809	0.66499
C65	0.76701	0.92671	0.34529
C66	0.64335	0.06894	0.76638
C67	0.76732	0.13225	0.36473
C68	0.64304	0.8634	0.74694
C69	0.76734	0.96726	0.4686
C70	0.64302	0.02839	0.64307
C71	0.76736	0.89398	0.33417
C72	0.643	0.10167	0.7775

C73	0.76352	0.17163	0.31141
C74	0.64684	0.82402	0.80026
C75	0.76349	0.02755	0.51495
C76	0.64687	0.9681	0.59672
C77	0.76352	0.79431	0.34113
C78	0.64684	0.20135	0.77054
C79	0.73789	0.21102	0.30613
C80	0.67246	0.78463	0.80554
C81	0.73787	0.01578	0.53728
C82	0.67249	0.97987	0.57439
C83	0.73791	0.76669	0.32408
C84	0.67245	0.22896	0.78759
C85	0.70515	0.03063	0.40011
C86	0.70521	0.96502	0.71156
C87	0.70514	0.03138	0.37799
C88	0.70522	0.96427	0.73368
C89	0.70511	0.06659	0.36625
C90	0.70525	0.92906	0.74542
C91	0.70519	0.13363	0.34389
C92	0.70517	0.86202	0.76778
C93	0.63788	0.26639	0.3244
C94	0.77248	0.72926	0.78727
C95	0.63793	0.96069	0.55584
O96	0.58311	0.20618	0.35869
O97	0.82725	0.78947	0.75299
O98	0.58318	0.93934	0.5086
O99	0.82718	0.05631	0.60307
O100	0.58317	0.84793	0.30021
O101	0.82719	0.14773	0.81147
H102	0.82146	0.09897	0.39154
H103	0.5889	0.89668	0.72013
H104	0.82156	0.9437	0.43855
H105	0.5888	0.05195	0.67312
H106	0.8216	0.95082	0.33743
H107	0.58876	0.04483	0.77425
H108	0.81948	0.15677	0.37233
H109	0.59088	0.83888	0.73934
H110	0.81949	0.94359	0.47706
H111	0.59087	0.05206	0.63461
H112	0.81948	0.89314	0.31811
H113	0.59088	0.10252	0.79357
H114	0.70514	0.05636	0.40869
H115	0.70522	0.93929	0.70298

H116	0.58786	0.2804	0.33843
H117	0.8225	0.71525	0.77325
H118	0.58793	0.93264	0.55584
N119	0.7052	0.16744	0.33262
N120	0.70515	0.82821	0.77905
C121	0.64347	0.39643	0.87555
C122	0.76689	0.59922	0.23612
C123	0.6434	0.52809	0.94668
C124	0.76696	0.46756	0.16499
C125	0.64335	0.56894	0.84529
C126	0.76701	0.42671	0.26638
C127	0.64304	0.3634	0.86473
C128	0.76732	0.63225	0.24694
C129	0.64302	0.52839	0.9686
C130	0.76734	0.46726	0.14307
C131	0.643	0.60167	0.83417
C132	0.76736	0.39398	0.2775
C133	0.64684	0.32402	0.81141
C134	0.76352	0.67163	0.30026
C135	0.64687	0.4681	0.01495
C136	0.76349	0.52755	0.09672
C137	0.64684	0.70135	0.84113
C138	0.76352	0.29431	0.27054
C139	0.67246	0.28463	0.80613
C140	0.73789	0.71102	0.30554
C141	0.67249	0.47987	0.03728
C142	0.73787	0.51578	0.07439
C143	0.67245	0.72896	0.82408
C144	0.73791	0.26669	0.28759
C145	0.70521	0.46502	0.90011
C146	0.70515	0.53063	0.21156
C147	0.70522	0.46427	0.87799
C148	0.70514	0.53138	0.23368
C149	0.70525	0.42906	0.86625
C150	0.70511	0.56659	0.24542
C151	0.70517	0.36202	0.84389
C152	0.70519	0.63363	0.26778
C153	0.77248	0.22926	0.8244
C154	0.63788	0.76639	0.28727
C155	0.77243	0.53497	0.05584
O156	0.82725	0.28947	0.85869
O157	0.58311	0.70618	0.25299
O158	0.82718	0.55631	0.0086

O159	0.58318	0.43934	0.10307
O160	0.82719	0.64773	0.80021
O161	0.58317	0.34793	0.31147
H162	0.5889	0.39668	0.89154
H163	0.82146	0.59897	0.22013
H164	0.5888	0.55195	0.93855
H165	0.82156	0.4437	0.17312
H166	0.58876	0.54483	0.83743
H167	0.8216	0.45082	0.27425
H168	0.59088	0.33888	0.87233
H169	0.81948	0.65677	0.23934
H170	0.59087	0.55206	0.97706
H171	0.81949	0.44359	0.13461
H172	0.59088	0.60252	0.81811
H173	0.81948	0.39314	0.29357
H174	0.70522	0.43929	0.90869
H175	0.70514	0.55636	0.20298
H176	0.8225	0.21525	0.83843
H177	0.58786	0.7804	0.27325
H178	0.82243	0.56301	0.05584
N179	0.70515	0.32821	0.83262
N180	0.7052	0.66744	0.27905
C181	0.64347	0.89643	0.37555
C182	0.76689	0.09922	0.73612
C183	0.6434	0.02809	0.44668
C184	0.76696	0.96756	0.66499
C185	0.64335	0.06894	0.34529
C186	0.76701	0.92671	0.76638
C187	0.64304	0.8634	0.36473
C188	0.76732	0.13225	0.74694
C189	0.64302	0.02839	0.4686
C190	0.76734	0.96726	0.64307
C191	0.643	0.10167	0.33417
C192	0.76736	0.89398	0.7775
C193	0.64684	0.82402	0.31141
C194	0.76352	0.17163	0.80026
C195	0.64687	0.9681	0.51495
C196	0.76349	0.02755	0.59672
C197	0.64684	0.20135	0.34113
C198	0.76352	0.79431	0.77054
C199	0.67246	0.78463	0.30613
C200	0.73789	0.21102	0.80554
C201	0.67249	0.97987	0.53728

C202	0.73787	0.01578	0.57439
C203	0.67245	0.22896	0.32408
C204	0.73791	0.76669	0.78759
C205	0.70521	0.96502	0.40011
C206	0.70515	0.03063	0.71156
C207	0.70522	0.96427	0.37799
C208	0.70514	0.03138	0.73368
C209	0.70525	0.92906	0.36625
C210	0.70511	0.06659	0.74542
C211	0.70517	0.86202	0.34389
C212	0.70519	0.13363	0.76778
C213	0.77248	0.72926	0.3244
C214	0.63788	0.26639	0.78727
C215	0.77243	0.03497	0.55584
O216	0.82725	0.78947	0.35869
O217	0.58311	0.20618	0.75299
O218	0.82718	0.05631	0.5086
O219	0.58318	0.93934	0.60307
O220	0.82719	0.14773	0.30021
O221	0.58317	0.84793	0.81147
H222	0.5889	0.89668	0.39154
H223	0.82146	0.09897	0.72013
H224	0.5888	0.05195	0.43855
H225	0.82156	0.9437	0.67312
H226	0.58876	0.04483	0.33743
H227	0.8216	0.95082	0.77425
H228	0.59088	0.83888	0.37233
H229	0.81948	0.15677	0.73934
H230	0.59087	0.05206	0.47706
H231	0.81949	0.94359	0.63461
H232	0.59088	0.10252	0.31811
H233	0.81948	0.89314	0.79357
H234	0.70522	0.93929	0.40869
H235	0.70514	0.05636	0.70298
H236	0.8225	0.71525	0.33843
H237	0.58786	0.2804	0.77325
H238	0.82243	0.06301	0.55584
N239	0.70515	0.82821	0.33262
N240	0.7052	0.16744	0.77905
C241	0.29028	0.39895	0.12155
C242	0.4137	0.60175	0.76098
C243	0.2902	0.53062	0.05042
C244	0.41377	0.47009	0.83211

C245	0.29015	0.57147	0.15181
C246	0.41382	0.42923	0.73072
C247	0.28985	0.36593	0.13237
C248	0.41413	0.63477	0.75016
C249	0.28982	0.53092	0.0285
C250	0.41415	0.46978	0.85403
C251	0.28981	0.60419	0.16293
C252	0.41416	0.39651	0.7196
C253	0.29365	0.32655	0.18569
C254	0.41032	0.67416	0.69684
C255	0.29367	0.47063	0.98215
C256	0.4103	0.53008	0.90038
C257	0.29364	0.70387	0.15597
C258	0.41033	0.29683	0.72656
C259	0.31927	0.28716	0.19097
C260	0.3847	0.71355	0.69156
C261	0.31929	0.4824	0.95982
C262	0.38468	0.5183	0.92271
C263	0.31926	0.73149	0.17302
C264	0.38472	0.26921	0.70951
C265	0.35201	0.46755	0.09699
C266	0.35196	0.53316	0.78554
C267	0.35202	0.4668	0.11911
C268	0.35195	0.5339	0.76342
C269	0.35205	0.43159	0.13085
C270	0.35192	0.56912	0.75168
C271	0.35198	0.36454	0.15321
C272	0.352	0.63616	0.72932
C273	0.41929	0.23179	0.1727
C274	0.28468	0.76891	0.70983
C275	0.41924	0.53749	0.94126
O276	0.47406	0.29199	0.13841
O277	0.22992	0.70871	0.74412
O278	0.47398	0.55883	0.9885
O279	0.22999	0.44187	0.89403
O280	0.47399	0.65025	0.19689
O281	0.22998	0.35045	0.68563
H282	0.2357	0.3992	0.10556
H283	0.46827	0.6015	0.77697
H284	0.23561	0.55447	0.05855
H285	0.46836	0.44623	0.82398
H286	0.23557	0.54735	0.15967
H287	0.4684	0.45335	0.72285

H288	0.23769	0.34141	0.12477
H289	0.46629	0.6593	0.75776
H290	0.23768	0.55458	0.02004
H291	0.4663	0.44612	0.86249
H292	0.23769	0.60504	0.17899
H293	0.46628	0.39566	0.70353
H294	0.35202	0.44182	0.08841
H295	0.35195	0.55888	0.79412
H296	0.46931	0.21778	0.15867
H297	0.23466	0.78293	0.72386
H298	0.46923	0.56553	0.94126
N299	0.35196	0.33073	0.16448
N300	0.35201	0.66997	0.71805
C301	0.29028	0.89895	0.62155
C302	0.4137	0.10175	0.26098
C303	0.2902	0.03062	0.55042
C304	0.41377	0.97009	0.33211
C305	0.29015	0.07147	0.65181
C306	0.41382	0.92923	0.23072
C307	0.28985	0.86593	0.63237
C308	0.41413	0.13477	0.25016
C309	0.28982	0.03092	0.5285
C310	0.41415	0.96978	0.35403
C311	0.28981	0.10419	0.66293
C312	0.41416	0.89651	0.2196
C313	0.29365	0.82655	0.68569
C314	0.41032	0.17416	0.19684
C315	0.29367	0.97063	0.48215
C316	0.4103	0.03008	0.40038
C317	0.29364	0.20387	0.65597
C318	0.41033	0.79683	0.22656
C319	0.31927	0.78716	0.69097
C320	0.3847	0.21355	0.19156
C321	0.31929	0.9824	0.45982
C322	0.38468	0.0183	0.42271
C323	0.31926	0.23149	0.67302
C324	0.38472	0.76921	0.20951
C325	0.35201	0.96755	0.59699
C326	0.35196	0.03316	0.28554
C327	0.35202	0.9668	0.61911
C328	0.35195	0.0339	0.26342
C329	0.35205	0.93159	0.63085
C330	0.35192	0.06912	0.25168

C331	0.35198	0.86454	0.65321
C332	0.352	0.13616	0.22932
C333	0.41929	0.73179	0.6727
C334	0.28468	0.26891	0.20983
C335	0.41924	0.03749	0.44126
O336	0.47406	0.79199	0.63841
O337	0.22992	0.20871	0.24412
O338	0.47398	0.05883	0.4885
O339	0.22999	0.94187	0.39403
O340	0.47399	0.15025	0.69689
O341	0.22998	0.85045	0.18563
H342	0.2357	0.8992	0.60556
H343	0.46827	0.1015	0.27697
H344	0.23561	0.05447	0.55855
H345	0.46836	0.94623	0.32398
H346	0.23557	0.04735	0.65967
H347	0.4684	0.95335	0.22285
H348	0.23769	0.84141	0.62477
H349	0.46629	0.1593	0.25776
H350	0.23768	0.05458	0.52004
H351	0.4663	0.94612	0.36249
H352	0.23769	0.10504	0.67899
H353	0.46628	0.89566	0.20353
H354	0.35202	0.94182	0.58841
H355	0.35195	0.05888	0.29412
H356	0.46931	0.71778	0.65867
H357	0.23466	0.28293	0.22386
H358	0.46923	0.06553	0.44126
N359	0.35196	0.83073	0.66448
N360	0.35201	0.16997	0.21805
C361	0.4137	0.60175	0.12155
C362	0.29028	0.39895	0.76098
C363	0.41377	0.47009	0.05042
C364	0.2902	0.53062	0.83211
C365	0.41382	0.42923	0.15181
C366	0.29015	0.57147	0.73072
C367	0.41413	0.63477	0.13237
C368	0.28985	0.36593	0.75016
C369	0.41415	0.46978	0.0285
C370	0.28982	0.53092	0.85403
C371	0.41416	0.39651	0.16293
C372	0.28981	0.60419	0.7196
C373	0.41032	0.67416	0.18569

C374	0.29365	0.32655	0.69684
C375	0.4103	0.53008	0.98215
C376	0.29367	0.47063	0.90038
C377	0.41033	0.29683	0.15597
C378	0.29364	0.70387	0.72656
C379	0.3847	0.71355	0.19097
C380	0.31927	0.28716	0.69156
C381	0.38468	0.5183	0.95982
C382	0.31929	0.4824	0.92271
C383	0.38472	0.26921	0.17302
C384	0.31926	0.73149	0.70951
C385	0.35196	0.53316	0.09699
C386	0.35201	0.46755	0.78554
C387	0.35195	0.5339	0.11911
C388	0.35202	0.4668	0.76342
C389	0.35192	0.56912	0.13085
C390	0.35205	0.43159	0.75168
C391	0.352	0.63616	0.15321
C392	0.35198	0.36454	0.72932
C393	0.28468	0.76891	0.1727
C394	0.41929	0.23179	0.70983
C395	0.28473	0.46321	0.94126
O396	0.22992	0.70871	0.13841
O397	0.47406	0.29199	0.74412
O398	0.22999	0.44187	0.9885
O399	0.47398	0.55883	0.89403
O400	0.22998	0.35045	0.19689
O401	0.47399	0.65025	0.68563
H402	0.46827	0.6015	0.10556
H403	0.2357	0.3992	0.77697
H404	0.46836	0.44623	0.05855
H405	0.23561	0.55447	0.82398
H406	0.4684	0.45335	0.15967
H407	0.23557	0.54735	0.72285
H408	0.46629	0.6593	0.12477
H409	0.23769	0.34141	0.75776
H410	0.4663	0.44612	0.02004
H411	0.23768	0.55458	0.86249
H412	0.46628	0.39566	0.17899
H413	0.23769	0.60504	0.70353
H414	0.35195	0.55888	0.08841
H415	0.35202	0.44182	0.79412
H416	0.23466	0.78293	0.15867

H417	0.46931	0.21778	0.72386
H418	0.23474	0.43517	0.94126
N419	0.35201	0.66997	0.16448
N420	0.35196	0.33073	0.71805
C421	0.4137	0.10175	0.62155
C422	0.29028	0.89895	0.26098
C423	0.41377	0.97009	0.55042
C424	0.2902	0.03062	0.33211
C425	0.41382	0.92923	0.65181
C426	0.29015	0.07147	0.23072
C427	0.41413	0.13477	0.63237
C428	0.28985	0.86593	0.25016
C429	0.41415	0.96978	0.5285
C430	0.28982	0.03092	0.35403
C431	0.41416	0.89651	0.66293
C432	0.28981	0.10419	0.2196
C433	0.41032	0.17416	0.68569
C434	0.29365	0.82655	0.19684
C435	0.4103	0.03008	0.48215
C436	0.29367	0.97063	0.40038
C437	0.41033	0.79683	0.65597
C438	0.29364	0.20387	0.22656
C439	0.3847	0.21355	0.69097
C440	0.31927	0.78716	0.19156
C441	0.38468	0.0183	0.45982
C442	0.31929	0.9824	0.42271
C443	0.38472	0.76921	0.67302
C444	0.31926	0.23149	0.20951
C445	0.35196	0.03316	0.59699
C446	0.35201	0.96755	0.28554
C447	0.35195	0.0339	0.61911
C448	0.35202	0.9668	0.26342
C449	0.35192	0.06912	0.63085
C450	0.35205	0.93159	0.25168
C451	0.352	0.13616	0.65321
C452	0.35198	0.86454	0.22932
C453	0.28468	0.26891	0.6727
C454	0.41929	0.73179	0.20983
C455	0.28473	0.96321	0.44126
O456	0.22992	0.20871	0.63841
O457	0.47406	0.79199	0.24412
O458	0.22999	0.94187	0.4885
O459	0.47398	0.05883	0.39403

O460	0.22998	0.85045	0.69689
O461	0.47399	0.15025	0.18563
H462	0.46827	0.1015	0.60556
H463	0.2357	0.8992	0.27697
H464	0.46836	0.94623	0.55855
H465	0.23561	0.05447	0.32398
H466	0.4684	0.95335	0.65967
H467	0.23557	0.04735	0.22285
H468	0.46629	0.1593	0.62477
H469	0.23769	0.84141	0.25776
H470	0.4663	0.94612	0.52004
H471	0.23768	0.05458	0.36249
H472	0.46628	0.89566	0.67899
H473	0.23769	0.10504	0.20353
H474	0.35195	0.05888	0.58841
H475	0.35202	0.94182	0.29412
H476	0.23466	0.28293	0.65867
H477	0.46931	0.71778	0.22386
H478	0.23474	0.93517	0.44126
N479	0.35201	0.16997	0.66448
N480	0.35196	0.83073	0.21805
C481	0.70518	0.49783	0.86731
C482	0.70518	0.49783	0.24437
C483	0.70518	0.49783	0.91154
C484	0.70518	0.49783	0.20013
C485	0.70518	0.49783	0.93501
C486	0.70518	0.49783	0.17666
C487	0.70518	0.49783	0.97972
C488	0.70518	0.49783	0.13195
H489	0.70518	0.49783	0.85015
H490	0.70518	0.49783	0.26152
N491	0.70518	0.49783	0.00226
N492	0.70518	0.49783	0.10941
C493	0.70518	-0.00217	0.36731
C494	0.70518	-0.00217	0.74437
C495	0.70518	-0.00217	0.41154
C496	0.70518	-0.00217	0.70013
C497	0.70518	-0.00217	0.43501
C498	0.70518	-0.00217	0.67666
C499	0.70518	-0.00217	0.47972
C500	0.70518	-0.00217	0.63195
H501	0.70518	-0.00217	0.35015
H502	0.70518	-0.00217	0.76152

N503	0.70518	-0.00217	0.50226
N504	0.70518	-0.00217	0.60941
C505	0.35199	0.50035	0.12979
C506	0.35199	0.50035	0.75273
C507	0.35199	0.50035	0.08556
C508	0.35199	0.50035	0.79697
C509	0.35199	0.50035	0.06209
C510	0.35199	0.50035	0.82044
C511	0.35199	0.50035	0.01738
C512	0.35199	0.50035	0.86515
H513	0.35199	0.50035	0.14695
H514	0.35199	0.50035	0.73558
N515	0.35199	0.50035	0.99484
N516	0.35199	0.50035	0.88769
C517	0.35199	0.00035	0.62979
C518	0.35199	0.00035	0.25273
C519	0.35199	0.00035	0.58556
C520	0.35199	0.00035	0.29697
C521	0.35199	0.00035	0.56209
C522	0.35199	0.00035	0.32044
C523	0.35199	0.00035	0.51738
C524	0.35199	0.00035	0.36515
H525	0.35199	0.00035	0.64695
H526	0.35199	0.00035	0.23558
N527	0.35199	0.00035	0.49484
N528	0.35199	0.00035	0.38769

Geometrical coordinates (in fractional coordinate) of PI-COF- TT optimized by VASP

PI-COF-TT: Space group: P1			
a=36.7143 Å b=6.8764 Å c=63.5910 Å			
α=90° β=90° γ=90°			
**Atom**	**x**	**y**	**z**
N1	-0.5	-0.75	0.12833
N5	-0.5	-0.75	-0.24833
N33	-0.5	-0.75	-0.00577
N37	-0.5	-0.75	-0.11423
C9	-0.5	-0.75	0.08582

C13	-0.5	-0.75	-0.20582
C33	-0.5	-0.75	0.06222
C37	-0.5	-0.75	-0.18222
C57	-0.5	-0.75	0.01707
C61	-0.5	-0.75	-0.13708
N9	-0.53249	-0.75	0.09584
N13	-0.46751	-0.75	-0.21584
N25	-0.66865	-0.75	0.16288
N29	-0.33135	-0.75	-0.28288
C1	-0.53126	-0.75	0.11709
C5	-0.46874	-0.75	-0.23709
C25	-0.56667	-0.75	0.12889
C29	-0.43333	-0.75	-0.24889
C49	-0.63439	-0.75	0.15146
C53	-0.36561	-0.75	-0.27146
C73	-0.60044	-0.75	0.11845
C77	-0.39956	-0.75	-0.23845
C81	-0.46745	-0.75	0.05055
C85	-0.53255	-0.75	-0.17055
C89	-0.4321	-0.75	0.151
C93	-0.5679	-0.75	-0.271
C121	-0.63352	-0.75	0.12927
C125	-0.36648	-0.75	-0.24927
C129	-0.46714	-0.75	0.0286
C133	-0.53286	-0.75	-0.14861
C137	-0.39934	-0.75	0.16213
C141	-0.60066	-0.75	-0.28213
C169	-0.67252	-0.75	0.1854
C173	-0.32748	-0.75	-0.3054
C177	-0.53184	-0.75	-0.01896
C181	-0.46816	-0.75	-0.10104
C185	-0.29564	-0.75	0.15356
C189	-0.70437	-0.75	-0.27356
C217	-0.71254	-0.75	0.19019
C221	-0.28746	-0.75	-0.31019
C225	-0.51901	-0.75	-0.04136
C229	-0.48099	-0.75	-0.07864
C233	-0.26845	-0.75	0.17118
C237	-0.73155	-0.75	-0.29118
C265	-0.26966	-0.75	-0.32966
C269	-0.23034	-0.75	-0.29034
C273	-0.46068	-0.75	-0.06
O1	-0.71152	-0.75	0.13485

O5	-0.28848	-0.75	-0.25485
O9	-0.43651	-0.75	-0.01318
O13	-0.56349	-0.75	-0.10682
O17	-0.35197	-0.75	0.19834
O21	-0.64803	-0.75	-0.31834
H1	-0.60079	-0.75	0.1013
H5	-0.39921	-0.75	-0.2213
H9	-0.44155	-0.75	0.05895
H13	-0.55845	-0.75	-0.17895
H17	-0.45766	-0.75	0.15975
H21	-0.54235	-0.75	-0.27975
H49	-0.65878	-0.75	0.12037
H53	-0.34122	-0.75	-0.24037
H57	-0.44117	-0.75	0.02042
H61	-0.55883	-0.75	-0.14042
H65	-0.40005	-0.75	0.1792
H69	-0.59995	-0.75	-0.2992
H97	-0.28453	-0.75	-0.34453
H101	-0.21547	-0.75	-0.27547
H105	-0.43094	-0.75	-0.06
□	□	□	□

Ni-H_2O complex optimized by Dmol^3

C	2.32280000	-3.21480000	-0.98210000
C	3.61690000	-2.73440000	-0.68230000
C	3.75340000	-1.42340000	-0.24620000
C	2.62020000	-0.59990000	-0.11780000
N	1.34370000	-1.08540000	-0.39070000
C	1.23480000	-2.37430000	-0.81360000
C	2.61670000	0.77160000	0.31340000
C	3.75000000	1.58510000	0.48330000
C	3.60950000	2.89530000	0.91920000
C	2.31000000	3.38360000	1.18090000
C	1.22250000	2.55010000	0.98030000
N	1.33560000	1.26110000	0.55380000

H	2.16500000	-4.22870000	-1.35120000
H	4.49000000	-3.37990000	-0.78640000
H	4.73470000	-1.02190000	0.01120000
H	0.21930000	-2.70990000	-1.02760000
H	4.73650000	1.17740000	0.25720000
H	4.48330000	3.53240000	1.05910000
H	2.14830000	4.39580000	1.55290000
H	0.20410000	2.88650000	1.17960000
C	-2.48690000	-3.16000000	1.23920000
C	-3.76710000	-2.72150000	0.83860000
C	-3.89580000	-1.44770000	0.30210000
C	-2.76580000	-0.62470000	0.16280000
N	-1.50400000	-1.07070000	0.53310000
C	-1.39870000	-2.31870000	1.06920000
C	-2.75830000	0.71550000	-0.36130000
C	-3.89410000	1.47780000	-0.68490000
C	-3.75000000	2.75910000	-1.19870000
C	-2.44660000	3.27120000	-1.37830000
C	-1.36000000	2.48690000	-1.03090000
N	-1.47780000	1.22690000	-0.53020000
H	-2.33860000	-4.13610000	1.70250000
H	-4.63890000	-3.36710000	0.95410000
H	-4.86820000	-1.07740000	-0.02470000
H	-0.39810000	-2.59650000	1.40460000
H	-4.88550000	1.05400000	-0.51800000
H	-4.62550000	3.35660000	-1.45500000
H	-2.27970000	4.26290000	-1.80110000
H	-0.33720000	2.84270000	-1.16430000
Ni	-0.07630000	0.08960000	0.06590000

O	1.21140000	-1.32230000	2.83750000
H	0.71710000	-0.66740000	2.30210000
H	2.04990000	-1.39590000	2.35210000

Ni-CO_2 complex optimized by Dmol^3

C	2.37260000	-3.23630000	-1.18160000
C	3.67060000	-2.75630000	-0.89990000
C	3.80980000	-1.45940000	-0.42460000
C	2.67620000	-0.65240000	-0.23210000
N	1.39680000	-1.13780000	-0.48420000
C	1.28400000	-2.41040000	-0.95700000
C	2.67800000	0.70380000	0.24370000
C	3.81320000	1.51340000	0.41680000
C	3.67970000	2.80590000	0.90390000
C	2.38630000	3.28110000	1.21170000
C	1.29640000	2.45190000	1.00300000
N	1.40360000	1.18190000	0.52670000
H	2.21230000	-4.23560000	-1.58840000
H	4.54510000	-3.38850000	-1.05580000
H	4.79610000	-1.05800000	-0.18730000
H	0.26660000	-2.73720000	-1.17500000
H	4.79450000	1.11730000	0.15240000
H	4.55500000	3.44020000	1.04720000
H	2.23060000	4.27890000	1.62370000
H	0.28130000	2.77970000	1.23140000
C	-2.39700000	-3.26310000	1.18170000
C	-3.69460000	-2.78850000	0.88980000
C	-3.83600000	-1.49040000	0.41930000

C	-2.70490000	-0.67460000	0.24680000
N	-1.42680000	-1.15300000	0.51190000
C	-1.31150000	-2.42850000	0.97210000
C	-2.71160000	0.68990000	-0.20600000
C	-3.85250000	1.48870000	-0.39230000
C	-3.72330000	2.79410000	-0.84680000
C	-2.42820000	3.29200000	-1.11050000
C	-1.33290000	2.47380000	-0.89100000
N	-1.43600000	1.19120000	-0.44480000
H	-2.23460000	-4.26480000	1.58150000
H	-4.56690000	-3.42680000	1.03410000
H	-4.82180000	-1.09400000	0.17200000
H	-0.29340000	-2.75610000	1.18730000
H	-4.83620000	1.07290000	-0.16900000
H	-4.60300000	3.41990000	-1.00020000
H	-2.27510000	4.29990000	-1.49800000
H	-0.31770000	2.81490000	-1.09660000
Ni	-0.01550000	0.02170000	0.03590000
C	0.10810000	0.20040000	-3.04430000
O	-0.62050000	-0.71370000	-3.17330000
O	0.84190000	1.11990000	-3.00990000

Cartesian coordinates of Ni-COF complex (NiL_3 near triazine) with PI-COF-TT data omitted

N	23.66320000	20.90610000	-1.06200000
N	25.43350000	22.74130000	-0.37640000
C	26.16970000	23.76290000	0.10030000
C	26.51970000	24.86570000	-0.67570000

C	26.05920000	24.93060000	-1.99750000
C	25.27900000	23.88800000	-2.49350000
C	24.98830000	22.79460000	-1.66530000
C	24.19180000	21.63790000	-2.08560000
C	23.99270000	21.27440000	-3.42440000
C	23.22360000	20.15170000	-3.72120000
C	22.68210000	19.40360000	-2.66710000
C	22.93740000	19.80960000	-1.35910000
N	23.68330000	19.67640000	1.75250000
N	25.95340000	19.82170000	0.41310000
C	26.97250000	19.94010000	-0.46010000
C	27.68570000	18.84470000	-0.94080000
C	27.31220000	17.56060000	-0.52100000
C	26.24260000	17.42610000	0.36180000
C	25.58430000	18.57430000	0.82480000
C	24.45280000	18.54910000	1.75800000
C	24.17220000	17.47710000	2.61760000
C	23.07550000	17.55700000	3.47360000
C	22.28790000	18.71670000	3.46050000
C	22.63520000	19.75140000	2.59520000
N	23.11660000	22.69780000	1.40410000
N	25.34850000	21.92430000	2.59050000
C	26.36270000	21.30680000	3.22750000
C	26.67400000	21.55080000	4.56300000
C	25.88540000	22.45860000	5.28160000
C	24.82140000	23.08690000	4.63730000
C	24.57560000	22.80680000	3.28590000
C	23.47830000	23.40410000	2.51330000
C	22.84740000	24.60810000	2.85480000

C	21.80860000	25.08640000	2.05690000
C	21.43040000	24.34620000	0.92890000
C	22.12080000	23.17240000	0.63200000
Ni	24.52950000	21.29210000	0.78540000
H	26.49270000	23.66570000	1.13850000
H	27.14050000	25.65470000	-0.25070000
H	26.29440000	25.79060000	-2.62600000
H	24.87990000	23.92670000	-3.50690000
H	24.45760000	21.85500000	-4.22100000
H	23.05660000	19.85560000	-4.75780000
H	22.06390000	18.52530000	-2.85180000
H	22.55020000	19.25680000	-0.50180000
H	27.21810000	20.96000000	-0.76110000
H	28.52170000	18.99800000	-1.62360000
H	27.83840000	16.67950000	-0.88920000
H	25.90610000	16.43950000	0.68030000
H	24.82360000	16.60270000	2.62810000
H	22.84480000	16.73400000	4.15140000
H	21.41550000	18.81760000	4.10680000
H	22.06290000	20.67960000	2.55130000
H	26.93900000	20.60530000	2.62130000
H	27.52040000	21.04310000	5.02610000
H	26.09170000	22.66370000	6.33260000
H	24.17190000	23.77370000	5.17960000
H	23.18480000	25.17350000	3.72380000
H	21.31220000	26.02780000	2.29820000
H	20.61050000	24.66990000	0.28770000
H	21.87320000	22.56640000	-0.24140000

Cartesian coordinates of Ni-COF complex (NiL_3 away from triazine) with PI-COF-TT data omitted

N	20.79630000	19.81080000	2.37950000
N	22.95440000	18.35760000	2.84400000
C	23.92500000	17.44280000	3.03430000
C	24.42710000	17.12980000	4.29600000
C	23.88020000	17.77160000	5.41450000
C	22.85800000	18.69880000	5.22740000
C	22.41620000	18.98610000	3.92760000
C	21.35420000	19.95160000	3.61630000
C	20.93890000	20.96240000	4.49350000
C	19.91760000	21.82840000	4.10550000
C	19.33390000	21.67080000	2.84010000
C	19.81610000	20.65890000	2.01050000
N	20.89790000	19.17420000	-0.62990000
N	22.98820000	20.33070000	0.49950000
C	23.92960000	20.94690000	1.24080000
C	24.32980000	22.26210000	1.01520000
C	23.70560000	22.99140000	-0.00610000
C	22.72440000	22.36660000	-0.77270000
C	22.39310000	21.03010000	-0.51020000
C	21.38580000	20.27900000	-1.26490000
C	20.95380000	20.63090000	-2.55120000
C	19.99840000	19.84600000	-3.19250000
C	19.50230000	18.71410000	-2.53420000
C	19.98550000	18.41250000	-1.26240000
N	20.85550000	16.86930000	1.39470000
N	23.04900000	17.26110000	-0.01990000

C	24.05440000	17.59450000	-0.85140000
C	24.55410000	16.72510000	-1.81800000
C	23.96450000	15.46070000	-1.95360000
C	22.91370000	15.11360000	-1.10750000
C	22.48010000	16.02680000	-0.13580000
C	21.37830000	15.76130000	0.80000000
C	20.86990000	14.48200000	1.07190000
C	19.77930000	14.34680000	1.93020000
C	19.24910000	15.49220000	2.53690000
C	19.83230000	16.72720000	2.25640000
Ni	21.91970000	18.63220000	1.07850000
H	24.30440000	16.96570000	2.12910000
H	25.23390000	16.40310000	4.39720000
H	24.23710000	17.54230000	6.41910000
H	22.39070000	19.18550000	6.08370000
H	21.42910000	21.08250000	5.45990000
H	19.58700000	22.62270000	4.77520000
H	18.52140000	22.31730000	2.50280000
H	19.40650000	20.49850000	1.01110000
H	24.37510000	20.34360000	2.03380000
H	25.11340000	22.70660000	1.62830000
H	23.97380000	24.03240000	-0.19210000
H	22.20370000	22.91460000	-1.55760000
H	21.38450000	21.49780000	-3.05100000
H	19.65650000	20.10240000	-4.19550000
H	18.74740000	18.07640000	-2.99360000
H	19.63250000	17.54120000	-0.70900000
H	24.46240000	18.59830000	-0.71480000
H	25.38490000	17.03580000	-2.45230000

H	24.30880000	14.76420000	-2.71850000
H	22.41680000	14.14880000	-1.21050000
H	21.32730000	13.60440000	0.61470000
H	19.33930000	13.36760000	2.12480000
H	18.39000000	15.43100000	3.20580000
H	19.47130000	17.65290000	2.70930000

Cartesian coordinates of Ni-COF-CO_2 complex with PI-COF-TT data omitted

C	-9.35408800	2.89493400	-2.59864600
C	-9.15770700	2.11880800	-3.75369700
C	-9.38783500	0.74929700	-3.68674500
C	-9.80016000	0.16780500	-2.47992800
N	-10.00999000	0.93913600	-1.35529300
C	-9.78690200	2.27525800	-1.43634700
C	-10.02966000	-1.24622700	-2.25256900
C	-9.84934900	-2.26788200	-3.19429900
C	-10.16900600	-3.58139500	-2.86315900
C	-10.66168100	-3.85320800	-1.57568500
C	-10.78722200	-2.81031000	-0.66859500
N	-10.48074500	-1.52557300	-0.97844500
H	-9.16527000	3.96826100	-2.59549800
H	-8.83463400	2.58354200	-4.68549800
H	-9.25412800	0.12176600	-4.56749200
H	-9.94952800	2.83884400	-0.51792400
H	-9.46409800	-2.02732200	-4.18539300
H	-10.05477400	-4.38174400	-3.59460100
H	-10.96777500	-4.85606300	-1.27987900
H	-11.16978000	-2.97872300	0.33759100

C	-12.83509800	3.26992600	1.52047500
C	-12.69813600	3.15990800	2.91265200
C	-11.96718200	2.09507200	3.43246600
C	-11.38599100	1.16227400	2.56608500
N	-11.50191400	1.29343800	1.19922900
C	-12.22390000	2.32712800	0.70553000
C	-10.64511500	-0.01949100	2.96722900
C	-10.41183300	-0.42630300	4.28696400
C	-9.65866300	-1.56916400	4.53901600
C	-9.14959100	-2.29646900	3.44991900
C	-9.42507000	-1.86057000	2.16113000
N	-10.15589200	-0.74659700	1.90110900
H	-13.43159000	4.06259400	1.06996000
H	-13.16475500	3.88669000	3.57719800
H	-11.84769800	1.98057500	4.50932300
H	-12.32974100	2.35998500	-0.37799800
H	-10.82263600	0.15713600	5.11059700
H	-9.46865700	-1.89210800	5.56318700
H	-8.54170500	-3.18787300	3.59946300
H	-9.04625800	-2.39380300	1.28855200
Ni	-10.57234400	-0.02290600	0.18711200
C	-12.69350100	-0.51549100	-0.32266100
O	-12.95500600	-0.09570400	-1.40923100
O	-12.90166400	-1.10400200	0.70559200

Cartesian coordinates of Ni-COF- H_2O complex with PI-COF-TT data omitted

C	26.84270000	8.43050000	-0.67810000
C	27.57150000	8.66700000	-1.85490000

C	28.95610000	8.76530000	-1.77420000
C	29.59520000	8.62490000	-0.53530000
N	28.87800000	8.36340000	0.61010000
C	27.52780000	8.27800000	0.51920000
C	31.02450000	8.75010000	-0.30210000
C	31.97850000	9.10020000	-1.26680000
C	33.32450000	9.15080000	-0.92410000
C	33.69790000	8.83680000	0.39220000
C	32.70930000	8.52040000	1.31460000
N	31.39010000	8.48490000	0.99890000
H	25.75440000	8.37150000	-0.68660000
H	27.06360000	8.77940000	-2.81260000
H	29.54700000	8.94620000	-2.67100000
H	26.99810000	8.10000000	1.45520000
H	31.66170000	9.33440000	-2.28260000
H	34.07430000	9.42060000	-1.66720000
H	34.74290000	8.82980000	0.70130000
H	32.95980000	8.26420000	2.34420000
C	27.24410000	5.29240000	3.75890000
C	27.31200000	5.56440000	5.13600000
C	28.18760000	6.54910000	5.57950000
C	28.97890000	7.24910000	4.65670000
N	28.88570000	6.99840000	3.30400000
C	28.03220000	6.02710000	2.88530000
C	29.94750000	8.27980000	4.98460000
C	30.30060000	8.68210000	6.28110000
C	31.21870000	9.70890000	6.46220000
C	31.77100000	10.32850000	5.32780000
C	31.40270000	9.87850000	4.06790000

N	30.51240000	8.87210000	3.87500000
H	26.60030000	4.50480000	3.36820000
H	26.69550000	5.01020000	5.84410000
H	28.26180000	6.78040000	6.64190000
H	28.02610000	5.83330000	1.81160000
H	29.85550000	8.18620000	7.14250000
H	31.49860000	10.03000000	7.46510000
H	32.47590000	11.15470000	5.41900000
H	31.80560000	10.33790000	3.16450000
Ni	29.93230000	8.15680000	2.19580000
O	30.55340000	4.60400000	1.01960000
H	30.62090000	5.21430000	1.77260000
H	29.92600000	3.91230000	1.31420000

References

1. Kresse, G. Joubert, D. From ultrasoft pseudopotentials to the projector augmented wave method, Phys. Rev. B., 59 (1999) 1758-1775.

2. Kresse, G., Furthmüller, J., Efficiency of ab-initio total energy calculations for metals and semiconductors using a plane-wave basis set, Comput. Mater. Sci. 6 (1996) 15-50.

3. Perdew, J. P., Burke, K. & Ernzerhof, M. Generalized gradient approximation made simple, Phys. Rev. Lett., 77 (1996) 3865-3868.

4. Delley, B. An All-Electron Numerical Method for Solving the Local Density Functional for Polyatomic Molecules, J. Chem. Phys., 92 (1990) 508-517.

5. Delley, B. From Molecules to Solids with the DMol3 Approach, J. Chem. Phys., 113 (2000) 7756-7764.

6. Bergner, A.; Dolg, M.; Küchle, W.; Stoll, H.; Preuß, H. Ab Initio Energy-Adjusted Pseudopotentials for Elements of Groups 13-17, Mol. Phys., 80 (1993) 1431-1441.

7. Lee, C. S.; Hwang, T. S.; Wang, Y.; Peng, S. M.; Hwang, C. S. Charge Density and Bonding in Bis(diiminosuccinonitrilo)nickel, $Ni(C_4N_4H_2)_2$: A Combined Experimental and Theoretical Study, J. Phys. Chem., 100 (1996) 2934-2941

8. Lin, T.; Zhang, W. D.; Huang, J.; He, C. A DFT Study of the Amination of Fullerenes and Carbon Nanotubes: Reactivity and Curvature, J. Phys. Chem. B, 109 (2005) 13755-13760.

9. Fu, Y.; Zhu, X.; Huang, L.; Zhang, X.; Zhang, F.; Zhu, W., Azine-based covalent organic frameworks as metal-free visible light photocatalysts for CO_2 reduction with H_2O, Appl. Catal., B, 239 (2018) 46-51.

10. Lu, M.; Liu, J.; Li, Q.; Zhang, M.; Liu, M.; Wang, J.L.; Yuan, D.Q.; Lan, Y.Q., Rational Design of Crystalline Covalent Organic Frameworks for Efficient CO_2 Photoreduction with H_2O, Angew. Chem., Int. Ed., 36 (2019) 12392-12397.

11. Yang, S.; Hu, W.; Zhang, X.; He, P.; Pattengale, B.; Liu, C.; Cendejas, M.; Hermans, I.; Zhang, X.; Zhang, J.; Huang, J., 2D Covalent Organic Frameworks as Intrinsic Photocatalysts for Visible Light-Driven CO_2 Reduction, J. Am. Chem. Soc., 44 (2018) 14614-14618.

12. Zhong, H.; Hong, Z.; Yang, C.; Li, L.; Xu, Y.; Wang, X.; Wang, R., A Covalent Triazine-Based Framework Consisting of Donor–Acceptor Dyads for Visible-Light-Driven Photocatalytic CO2 Reduction, ChemSusChem, 19 (2019) 4493-4499.

13. Jiao, X.; Chen, Z.; Li, X.; Sun, Y.; Gao, S.; Yan, W.; Wang, C.; Zhang, Q.; Lin, Y.; Luo, Y.; Xie, Y., Defect-Mediated Electron–Hole Separation in One-Unit-Cell $ZnIn_2S_4$ Layers for Boosted Solar-Driven CO_2 Reduction, J. Am. Chem. Soc., 22 (2017) 7586-7594.

14. Shi, W.; Guo, X.; Cui, C.; Jiang, K.; Li, Z.; Qu, L.; Wang, J.C., Controllable synthesis of Cu_2O decorated WO_3 nanosheets with dominant (0 0 1) facets for photocatalytic CO_2 reduction under visible-light irradiation, Appl. Catal., B, 243 (2019) 236-242.

15. Wu, J.; Li, X.; Shi, W.; Ling, P.; Sun, Y.; Jiao, X.; Gao, S.; Liang, L.; Xu, J.; Yan, W.; Wang, C.; Xie, Y., Efficient Visible-Light-Driven CO_2 Reduction Mediated by

Defect-Engineered BiOBr Atomic Layers, Angew. Chem., Int. Ed., 28 (2018) 8855-8859.
